# Recurrent Acute Liver Failure in Children: A Systematic Review of Genetic Etiologies, Diagnostic Clues, and Liver Transplantation

**DOI:** 10.3390/genes17101225

**Published:** 2026-10-02

**Authors:** Patryk Lipiński, Piotr Socha, Irena Jankowska

**Affiliations:** 1 Third Department of Pediatrics, Center of Postgraduate Medical Education, 05-092 Dziekanów Leśny, Poland; 2 Department of Gastroenterology, Hepatology, Feeding Disorders and Pediatrics, The Children’s Memorial Health Institute, 04-730 Warsaw, Poland; p.socha@ipczd.pl (P.S.); i.m.jankowska@wp.pl (I.J.)

**Keywords:** recurrent liver failure, genomic diagnosis, hyperammonemia, metabolic decompensation, pediatric hepatology

## Abstract

Background: Recurrent acute liver failure (RALF) is a rare phenotype with heterogeneous inherited causes. Methods: We conducted a PRISMA 2020 systematic review of PubMed/MEDLINE and Web of Science Core Collection. Reports were classified into two non-combinable evidence sets: strict RALF, requiring at least two documented pediatric acute liver failure episodes with recovery of liver synthetic function between episodes, and contextual disease-specific evidence used only to inform broader natural history, treatment, or transplantation. Results: The searches identified 128 records; 30 duplicates were removed, and 98 records were screened. Forty-eight full texts were assessed, and 44 publications representing 41 study or cohort units were retained across both evidence sets. The evidence consisted predominantly of case reports, small case series, and retrospective cohorts, with inconsistent RALF definitions and incompletely reported follow-up. The five-group framework is a pragmatic organization of reported qualifying disorders rather than an exhaustive genetic classification. Conclusions: Genomic testing during a first unexplained severe episode is proposed to avoid waiting for recurrence, but most disorder-specific treatment and transplantation conclusions remain based on limited observational evidence and expert practice.

## 1. Introduction

Pediatric acute liver failure (PALF) is a rapidly evolving syndrome characterized by acute hepatocellular injury and coagulopathy in a child without known chronic liver disease. Encephalopathy may be absent or difficult to recognize in infants; therefore, commonly used PALF criteria include an international normalized ratio (INR) of at least 1.5 in the presence of hepatic encephalopathy or at least 2.0 without encephalopathy, not corrected by vitamin K [1]. Neonatal acute liver failure requires separate consideration because liver injury may begin in utero, encephalopathy is particularly difficult to assess, and physiological coagulation values differ from those of older children. A universal neonatal INR threshold has not been established; published approaches use higher thresholds, and the European Association for the Study of the Liver notes an INR greater than 4 in neonates or very young children when encephalopathy cannot be recognized [2,3].

In this review, RALF denotes two or more episodes fulfilling PALF criteria, with substantial recovery of hepatic synthetic function between episodes. This definition is narrower than recurrent acute liver injury, in which aminotransferase elevation may recur without clinically important coagulopathy, and it is also distinct from acute-on-chronic genetic liver disease, in which an acute decompensation occurs on a background of persistent fibrosis, portal hypertension, cholestasis, or chronically abnormal synthetic function. In practice, these categories can overlap: several monogenic disorders may cause recurrent hepatocellular injury, cholestatic crises, recurrent coagulopathy, or chronic liver disease with acute decompensation. For this reason, the term RALF should be applied after careful reconstruction of prior episodes, interval liver biochemistry, coagulation profile, imaging, and evidence of chronic liver disease.

RALF has a strong genetic basis: in a multicenter cohort of children with indeterminate PALF, a molecular diagnosis was established in 64% of children with recurrent episodes who underwent whole-exome sequencing (WES). Most identified conditions were inherited metabolic diseases (IMD) or other monogenic disorders affecting essential cellular processes [4,5]. Fever-triggered episodes followed by apparent recovery are especially characteristic of disorders such as *NBAS*-, *RINT1*-, *SCYL1*-, and *LARS1*-associated disease, but a similar recurrent pattern may also occur in selected urea-cycle, mitochondrial, aminoacyl-tRNA synthetase, and protein-stress disorders.

During the past decade, broader access to exome and genome sequencing has identified multiple inherited causes of PALF that were previously classified as indeterminate. A clinically important subset predisposes to recurrent crises triggered by fever, infection, fasting, increased protein intake, or another form of metabolic stress. These disorders can be grouped into five clinically useful groups: urea-cycle defects, disorders of energy metabolism, disorders of protein translation and cellular stress, disorders of ion homeostasis and channel-kinase signaling, and disorders of vesicular trafficking [4,5,6]. This clinically oriented framework helps prioritize biochemical tests, genomic analysis, anticipatory care, and disease-specific treatment.

The aim of this review is to summarize genetic causes of recurrent acute liver failure in children, highlight clinical and biochemical clues that should prompt metabolic and genomic testing, and discuss how molecular diagnosis informs emergency management, long-term surveillance, and liver transplantation decisions.

## 2. Materials and Methods

This systematic review was prepared in accordance with the Preferred Reporting Items for Systematic Reviews and Meta-Analyses (PRISMA) 2020 statement [7]. The review protocol was not prospectively registered. The review question was: in children with recurrent acute liver failure, which genetically determined disorders have been reported, which clinical and biochemical features support diagnosis, and what evidence informs acute management, surveillance, and liver transplantation?

### 2.1. Eligibility Criteria

Two evidence sets were prespecified and analyzed separately. The strict RALF set comprised individual patients younger than 18 years at first presentation with at least two PALF episodes and documented recovery of hepatic synthetic function between episodes. The contextual set comprised disease-specific cohorts or reports that did not establish strict RALF for every participant but directly informed disease characterization, broader natural history, treatment, or transplantation. Contextual studies were not included in RALF-specific patient denominators or proportions. Reviews, guidelines, and experimental studies were used only to contextualize interpretation and were not counted as primary clinical studies (Table S3).

#### Operational Definition of Strict RALF

An episode was classified as PALF when a child without known decompensated chronic liver disease had acute liver injury and coagulopathy not corrected by parenteral vitamin K: INR ≥ 2.0 without a requirement for encephalopathy, or INR ≥ 1.5 with hepatic encephalopathy. Historical episodes lacking an INR were not considered unequivocal PALF and were assigned to contextual evidence. Interval recovery required normalization of INR without plasma replacement or factor therapy and absence of ongoing clinical hepatic decompensation; normalization of aminotransferase activity or bilirubin was recorded but was not mandatory. Portal hypertension, established cirrhosis, or persistent synthetic dysfunction argued for acute-on-chronic disease rather than strict RALF. Patients with recurrent injury but only one documented PALF episode were retained only in the contextual set. Neonatal reports were judged using the same liver-based coagulopathy principle, with explicit consideration of gestational age, vitamin K deficiency, sepsis, and developmentally appropriate reference ranges.

Scope clarification: inherited cholestatic and bile-acid synthesis disorders were eligible only when a report documented at least two discrete PALF episodes with recovery of hepatic synthetic function between episodes. *HSD3B7* deficiency was not included because the retrieved literature described neonatal or childhood cholestasis, malabsorption, fibrosis, cirrhosis, or progressive liver failure rather than a qualifying recurrent PALF phenotype. It remains an important differential diagnosis of low-GGT cholestatic liver disease but is outside the strict population synthesized in this review.

### 2.2. Information Sources and Search Strategy

PubMed/MEDLINE and Web of Science Core Collection were searched from inception through 2 September 2026. The reproducible PubMed strategy combined terms for acute liver failure, recurrence, pediatric age, and genetic or metabolic disease; gene-specific terms were used only as a supplementary sensitivity search. The complete database-specific strategies, search dates, and record counts are provided in Supplementary Table S3. No language filter was applied at database level. Non-English reports were assessed from English abstracts and translated full text where accessible. Backward reference checking and forward citation tracking were performed for included reports and key reviews. Retraction and correction status was checked in the source databases. The strategy was not formally PRESS-reviewed and was not developed by an information specialist. Embase and Scopus were not searched because institutional access and export suitable for deduplication were unavailable during the review; this restriction may have reduced sensitivity and is acknowledged as a limitation.

### 2.3. Study Selection

**Figure 1:**
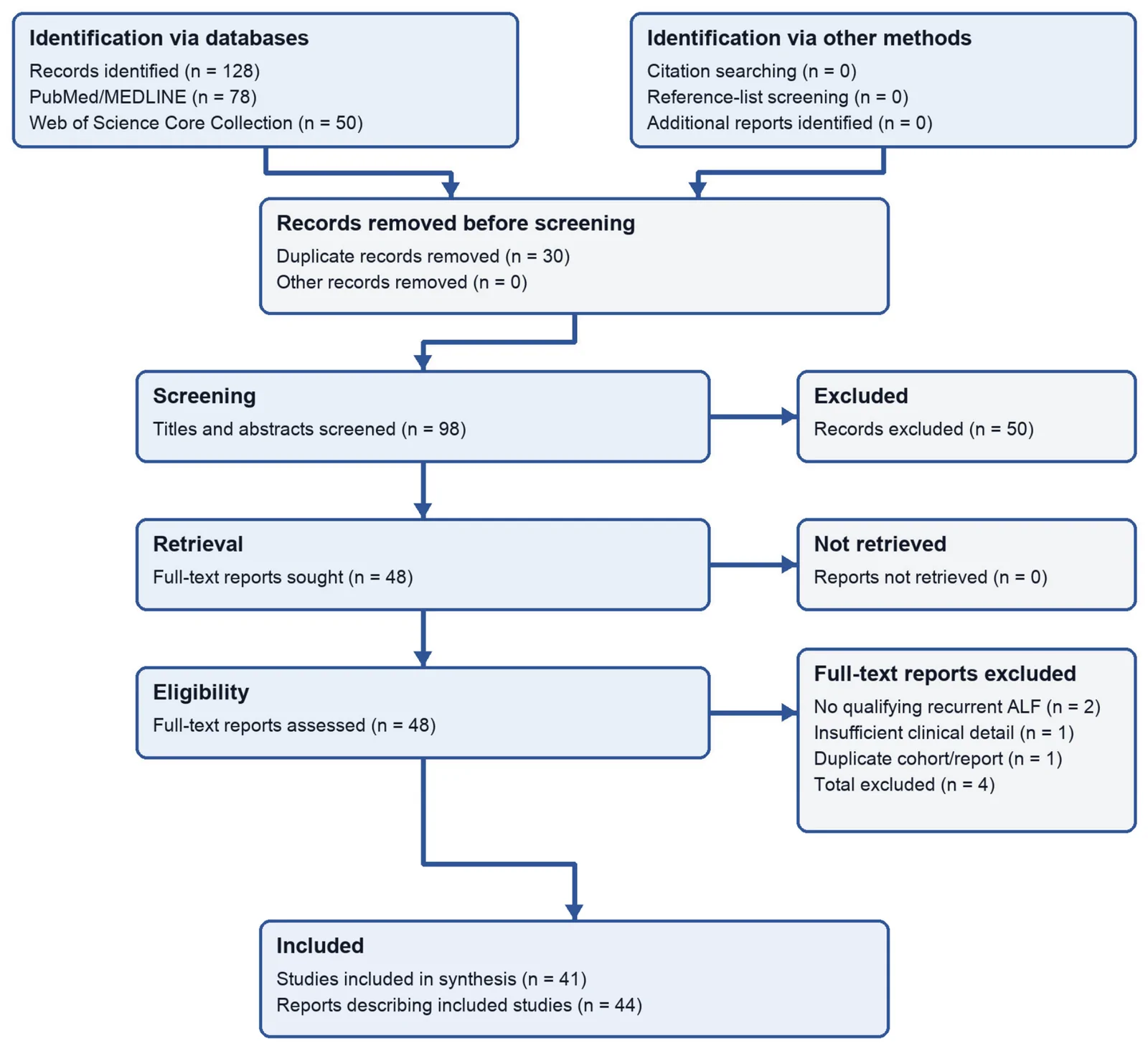
PRISMA 2020 flow diagram of study identification and selection.

P.L. and P.S. independently screened titles and abstracts and then assessed potentially eligible full-text reports. The reviewers independently determined inclusion and documented the primary reason for full-text exclusion. Disagreements were resolved through discussion; unresolved decisions were adjudicated by I.J. Multiple reports describing the same or overlapping patients were linked and treated as one study where possible. The study-selection process is shown in Figure 1. Reports were tagged at patient level as strict RALF, probable RALF with incomplete historical documentation, recurrent acute liver injury, acute-on-chronic disease, or contextual disease evidence. Inter-reviewer agreement statistics were not calculated. A written protocol was prepared before the final reconciliation but was not prospectively registered; the principal post hoc amendment was explicit separation of strict and contextual evidence in response to peer review.

### 2.4. Data Collection and Outcomes

Two reviewers independently extracted the gene, inheritance, age at onset, number and triggers of episodes, biochemical phenotype, interval recovery, extrahepatic manifestations, molecular findings, acute and preventive treatment, liver-transplantation indication, age at transplantation, graft and patient outcome, recurrence after transplantation, and duration of follow-up. Where reports overlapped, the most complete or most recent source was designated the primary report, and supplementary reports were used only to complete missing variables.

### 2.5. Critical Appraisal

Methodological quality was assessed independently by P.L. and P.S. using the Joanna Briggs Institute design-specific critical-appraisal checklists for case reports, case series, and cohort studies [8]. Appraisal informed interpretation but was not used as an exclusion criterion because the evidence base for ultra-rare disorders is necessarily dominated by descriptive studies. Particular attention was paid to patient ascertainment, completeness of phenotype and follow-up, confirmation of molecular diagnosis, transparent reporting of overlapping cohorts, and ascertainment of transplantation outcomes (Table S2).

### 2.6. Synthesis Methods

Because of marked clinical heterogeneity, non-comparative designs, overlapping cohorts, and incomplete outcome reporting, meta-analysis was not considered appropriate. Findings were synthesized narratively and grouped by the dominant clinical and biochemical pattern. Counts and proportions were reported only when the denominator was traceable; overlapping cohorts were not summed. Experimental findings were interpreted separately from clinical outcome evidence.

Because most of the available evidence comes from case reports and small retrospective series, we indicate throughout the review whether a statement is based on published guidance, supported mainly by observational data, or reflects the authors’ clinical synthesis. This distinction is intended to help readers judge the confidence that can be placed in individual recommendations and should not be interpreted as a formal GRADE assessment.

## 3. Results

### 3.1. Study Selection and Characteristics

The database searches identified 128 records (PubMed/MEDLINE, *n* = 78; Web of Science Core Collection, *n* = 50). After removal of 30 duplicate records, 98 titles and abstracts were screened; 50 were excluded. Forty-eight full texts were assessed, and four were excluded (non-qualifying recurrent ALF, *n* = 2; insufficient extractable clinical detail, *n* = 1; superseded overlapping cohort, *n* = 1). Forty-four publications representing 41 study or cohort units were retained across the strict and contextual evidence sets (Figure 1). These totals must not be interpreted as 41 independent participant populations because several publications contained partially overlapping patients. Most reports were case reports, small case series, or retrospective disease cohorts; no randomized or prospective comparative treatment or transplantation study was identified.

#### Evidence Set Interpretation

Only patient-level data meeting the operational definition were used for strict RALF statements. Broader cohort percentages—such as any liver involvement, at least one ALF episode, or transplant experience without documented recurrence—are labeled as contextual and are not pooled with strict RALF denominators. Because complete non-overlapping patient-level denominators could not be reconstructed for every gene, this review does not report new pooled gene-specific proportions. Quantitative estimates from individual cohorts are retained only with their source population explicitly stated.

Whenever patient-level information allowed, we determined how many individuals met the strict RALF definition. Studies that grouped recurrent PALF with recurrent hepatitis, a single episode of ALF, or cases without documented recovery between episodes were retained as contextual evidence, but their entire cohorts were not counted as strict RALF. For each disorder, Supplementary Table S3 therefore reports the minimum number of reliably classifiable strict-RALF cases, the broader contextual denominator, and, when a precise count was not possible, the reason.

### 3.2. Clinical Grouping of Inherited RALF

**Figure 2:**
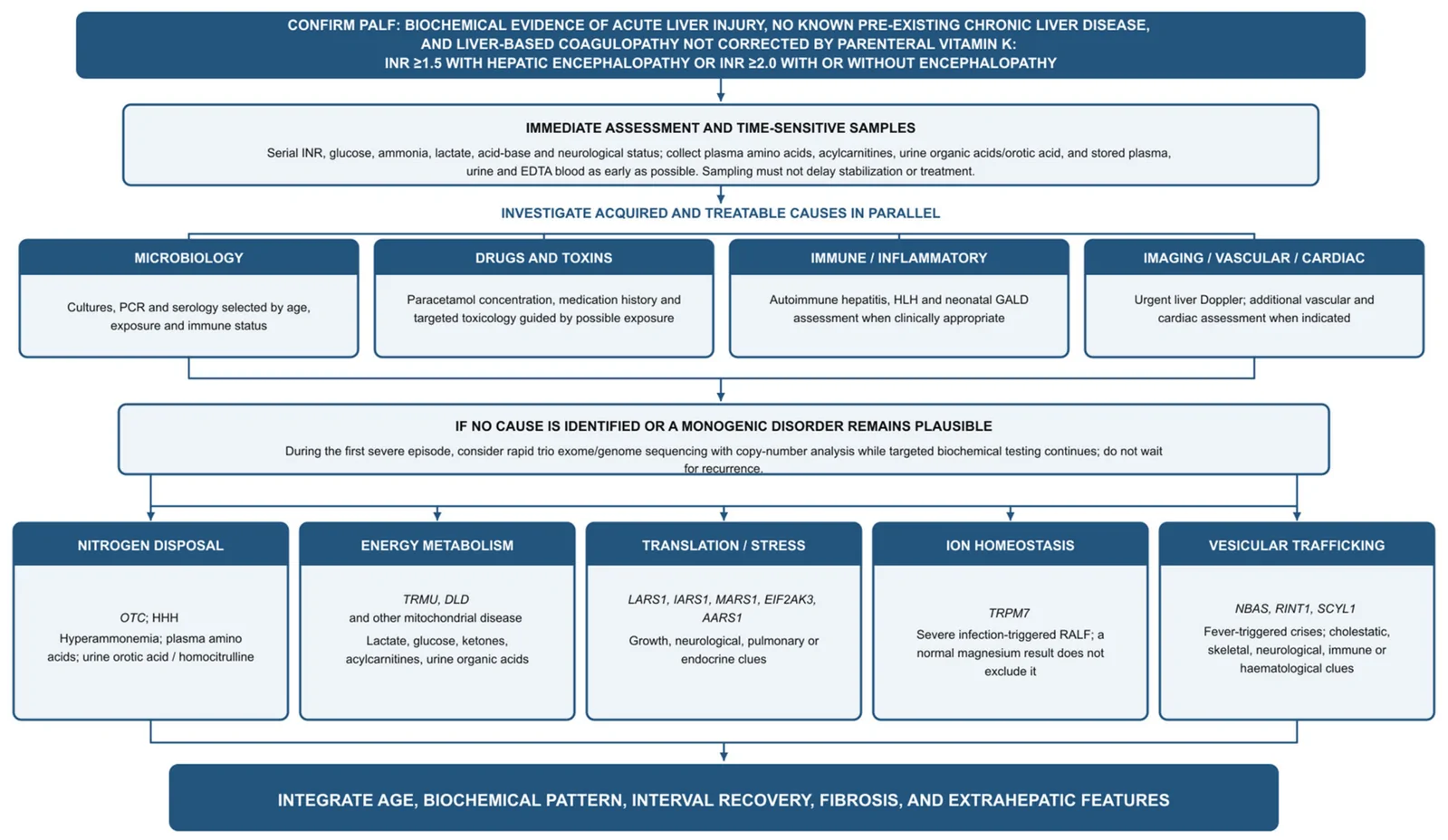
Author-proposed diagnostic framework for pediatric acute or recurrent liver failure. Confirmation and immediate assessment of PALF are followed by parallel evaluation of acquired and treatable causes. When the initial evaluation is unrevealing or a monogenic disorder remains plausible, rapid genomic testing is integrated with phenotype-directed biochemical investigation and multisystem assessment.

The pragmatic five-group classification is clinically useful because inherited disorders with different biochemical and cellular features can present with the same endpoint of acute hepatocellular dysfunction. The groups are distinguished by their typical triggers, biochemical abnormalities, extrahepatic features, and treatment priorities [6]. The classification is not exhaustive and does not exclude additional inherited causes. This framework is summarized in Figure 2.

#### 3.2.1. Urea-Cycle Disorders

Urea-cycle disorders primarily cause hyperammonemia, but acute hepatocellular injury and impaired hepatic synthetic function may dominate the initial presentation. Ornithine transcarbamylase (OTC) deficiency and hyperornithinemia–hyperammonemia–homocitrullinuria (HHH) syndrome are particularly relevant in RALF. Episodes may be precipitated by fever, infection, fasting, catabolism, or increased protein intake. Ammonia must therefore be measured urgently in every child with otherwise unexplained acute liver dysfunction, even when encephalopathy is absent or attributed to liver failure [9,10].

The discussion of OTC deficiency draws on strict RALF case reports and small pediatric series, supplemented by broader urea-cycle guidance. Ornithine transcarbamylase (OTC) deficiency is X-linked. Hemizygous boys with severe loss-of-function variants typically present in the first days of life with rapidly progressive hyperammonemia, poor feeding, vomiting, respiratory instability, lethargy, seizures, and hyperammonemic encephalopathy that may progress to coma and death. Coagulopathy and impaired hepatic synthetic function may accompany the crisis, whereas aminotransferase activity can initially be normal or only moderately increased; the dominant emergency is therefore hyperammonemia rather than primary massive hepatocellular necrosis. Boys with partial residual enzyme activity may instead present later, at any age, with episodic vomiting, encephalopathy, behavioral or neurological symptoms, or acute liver dysfunction precipitated by infection, fasting, surgery, or increased protein intake. In heterozygous girls, the phenotype ranges from asymptomatic to severe because of variable hepatic X-chromosome inactivation. An underrecognized female presentation consists of early hyperammonemia and coagulopathy followed by a delayed aminotransferase peak; ammonia elevation may be modest or transient relative to the apparent liver injury [9]. Plasma amino acids, blood ammonia, and urinary orotic acid should be obtained before prolonged protein restriction whenever feasible, because reduced substrate intake may lower orotic acid excretion and obscure the biochemical diagnosis. Molecular analysis of *OTC* is required when biochemical findings are equivocal. Acute management combines temporary protein interruption, high-energy intravenous glucose with or without lipids, nitrogen-scavenging therapy, arginine or citrulline as appropriate, and prompt extracorporeal ammonia removal when severe hyperammonemia fails to fall rapidly with medical treatment [10]. Liver transplantation (LTx) replaces the major hepatic enzyme deficit, prevents further hyperammonemic crises, permits major relaxation or discontinuation of protein restriction and nitrogen-scavenger therapy, and improves metabolic stability; it cannot reverse established brain injury or correct extrahepatic OTC deficiency. Referral should therefore be considered early in boys with neonatal-onset disease who survive the initial crisis and in any patient with recurrent or medically difficult-to-control hyperammonemia, poor metabolic control despite optimal therapy, severe dietary intolerance, impaired quality of life, or unacceptable risk of further neurological injury. LTx is ideally undertaken during metabolic stability and before irreversible neurological damage, often after infancy when surgical risk is lower, although timing must be individualized. An isolated episode of hepatic dysfunction that resolves with metabolic treatment does not by itself mandate urgent LTx [10,11].

Evidence for HHH syndrome in RALF is limited to rare strict or probable cases and is supplemented by contextual natural-history and transplantation data. HHH syndrome results from biallelic *SLC25A15* variants and impaired mitochondrial ornithine transport. The diagnostic biochemical triad comprises hyperornithinemia, hyperammonemia, and urinary homocitrulline, although the complete pattern may not be present in every patient. Protein aversion, recurrent vomiting after protein-rich meals, spasticity, pyramidal signs, cognitive impairment, and white-matter abnormalities provide additional clues [12]. Acute treatment follows the principles used for other urea-cycle decompensations; long-term therapy includes individualized protein restriction, adequate energy intake, nitrogen scavengers, and citrulline or arginine supplementation [12]. Neurological disease may progress despite conventional metabolic control. Limited observational evidence suggests that LTx can restore hepatic ureagenesis and prevent further metabolic crises, but referral should be individualized and ideally occur before irreversible neurological injury [13].

#### 3.2.2. Disorders of Energy Metabolism

Energy-metabolism disorders should be suspected when RALF is accompanied by persistent or recurrent lactic acidosis, hypoglycemia, cardiomyopathy, myopathy, or neurological abnormalities. Their hepatic phenotype may range from isolated aminotransferase elevation and hepatomegaly to steatosis, impaired synthetic function, and acute liver failure (ALF). Biochemical abnormalities are often most informative during decompensation and may normalize between episodes; samples for lactate, pyruvate, plasma amino acids, acylcarnitines, urinary organic acids, and ketones should therefore be obtained early, without delaying stabilization or genomic testing.

The evidence for TRMU deficiency combines strict or probable RALF cases with broader infantile liver-failure cohorts; spontaneous recovery is common and complicates attribution of treatment effects. Biallelic *TRMU* variants impair 2-thiolation of selected mitochondrial transfer RNAs and thereby compromise mitochondrial protein synthesis. Recent experimental work supports a direct link between deficient *TRMU*-dependent mitochondrial tRNA modification, impaired mitochondrial translation and respiratory-chain function, and the liver-predominant phenotype [14]. The typical presentation is an infant, often previously well, who develops poor feeding, vomiting, hypoglycemia, persistent lactic acidosis, hepatomegaly, cholestasis, coagulopathy, and rapidly progressive ALF [15,16]. Ascites, hyperammonemia, respiratory failure, renal dysfunction, or multiorgan failure may occur in severe disease [15]. In the largest international series, liver involvement was present in 60 of 62 individuals, and ALF occurred in 43. Recurrent liver-failure episodes were documented in 14 of 62 patients (22.6%), predominantly during the first five months of life; as many as five episodes occurred in two unrelated children. Apart from a later episode associated with hepatoblastoma, ALF was confined to the first year of life. Eleven patients underwent LTx [15]. Extrahepatic findings were less consistent and included developmental disability, hypotonia, seizures, cardiomyopathy, skeletal myopathy, respiratory involvement, and renal dysfunction; some may reflect secondary injury during critical illness rather than primary persistent mitochondrial disease. Most surviving non-transplanted infants improved after the first year of life, possibly because endogenous cysteine availability increases with maturation, but cirrhosis or persistent biochemical abnormalities have occasionally been reported [15].

Limited retrospective reports describe N-acetylcysteine and L-cysteine supplementation in TRMU deficiency, but the number of treated patients, formulations, routes, monitoring, and dose selection were heterogeneous. No standardized regimen or comparative efficacy evidence is available. Therefore, exact doses are not proposed here; use should be individualized by a metabolic specialist with monitoring for formulation-specific adverse effects.

The evidence for hepatic DLD deficiency is derived from disease-specific cohorts and selected recurrent or acute liver-failure cases, not all of which met strict RALF criteria. Dihydrolipoamide dehydrogenase (DLD) is the shared E3 component of the pyruvate dehydrogenase, 2-oxoglutarate dehydrogenase, and branched-chain 2-oxoacid dehydrogenase complexes and also participates in the glycine-cleavage system. DLD deficiency therefore produces overlapping hepatic, neurological, and myopathic phenotypes. The hepatic form may begin from the neonatal period to adulthood, typically with infection-, fasting-, surgery-, or dietary-stress-triggered nausea, vomiting, abdominal pain, hepatomegaly, hypoglycemia, lactic acidosis, marked ALT elevation, coagulopathy, hepatic encephalopathy, and recurrent ALF. Episodes may resolve completely, recur throughout life with decreasing frequency, or progress to liver failure. In the 2025 series of 52 patients with hepatic DLD deficiency, all had hepatic involvement; hypoglycemia and lactic acidosis were frequent, while neurological manifestations were uncommon in patients homozygous for the recurrent p.Gly229Cys variant but more frequent with another *DLD* variant [17]. Extrahepatic manifestations across the broader DLD spectrum include developmental disability, hypotonia, seizures, ataxia, optic involvement, behavioral disturbance, exercise intolerance, myalgia, weakness, rhabdomyolysis or hyperCKemia, and cardiomyopathy. Their presence is particularly important when assessing transplant candidacy.

During a *DLD*-related crisis, treatment focuses on rapid intravenous glucose delivery, avoidance of fasting, correction of hypoglycemia and acidosis, and intensive organ support. Lactate, acylcarnitines, plasma amino acids, and urinary organic acids may be unremarkable between episodes and are not uniformly diagnostic even during decompensation, so a normal interval metabolic screen does not exclude the disorder. Riboflavin, thiamine, carnitine, biotin, coenzyme Q10, and other supplements have been used, but responses are inconsistent, and no disease-specific regimen has high-quality evidence [17]. Long-term care requires an individualized emergency protocol and neurological, cardiac, ophthalmological, and muscular surveillance. Evidence for LTx is exceptionally limited: the largest hepatic cohort identified only one transplanted patient, who had good graft engraftment but poor systemic recovery and died from sepsis after transplantation [17,18]. LTx should therefore not be described as established prevention of hepatic crises. It may be considered as rescue therapy for otherwise irreversible liver failure only after careful exclusion of severe progressive extrahepatic mitochondrial disease and explicit assessment of perioperative catabolic risk; whether it improves long-term survival or prevents recurrent metabolic decompensation remains unknown.

#### 3.2.3. Disorders of Protein Translation and Cellular Stress

Disorders of cytosolic aminoacyl-tRNA synthetases and cellular stress responses connect impaired protein synthesis or proteostasis with tissue-selective vulnerability. They should be considered when recurrent, infection-triggered liver dysfunction coexists with poor growth, neurodevelopmental disability, hypotonia, anemia, interstitial lung disease, neonatal diabetes, or skeletal abnormalities. No single biochemical marker reliably identifies this group; early exome or genome sequencing is therefore often decisive [6].

For LARS1 deficiency, the literature comprises broader disease cohorts containing recurrent liver injury, ALF, and chronic liver disease, with strict RALF documented inconsistently. Biallelic *LARS1* variants cause infantile liver failure syndrome type 1. In the largest disease-specific cohort, recurrent ALT elevation and liver failure were commonly triggered by febrile illness and accompanied by growth restriction, microcytic anemia, hypoalbuminemia, hypotonia, seizures, and neurodevelopmental abnormalities [19]. Encephalopathic episodes may occur independently of biochemical liver failure and can resemble a metabolic stroke on magnetic resonance imaging. Functional studies show temperature-sensitive reduction of leucyl-transfer RNA aminoacylation, providing a plausible link between fever and decompensation. Management remains supportive and includes early treatment with antipyretic drugs, avoidance of fasting, adequate glucose delivery, and an individualized emergency protocol. Evidence for LTx is limited; transplantation may control liver crises but cannot be assumed to prevent neurological or other extrahepatic manifestations.

The association between *AARS1* and RALF remains emerging and is supported by one strict, functionally characterized case. Biallelic *AARS1* variants have also been associated with RALF. The evidence currently consists of a single functionally characterized patient with compound-heterozygous missense variants p.(Leu298Gln) and p.(Arg751Gly), reduced alanyl-tRNA synthetase activity in fibroblasts, microcephaly, developmental delay, and four episodes of infection- or fever-triggered liver dysfunction beginning at 2 years of age. Coagulopathy, hypoglycemia, marked aminotransferase elevation, and complete biochemical recovery within approximately 2 weeks were documented. This report extends the hepatic spectrum of cytosolic aminoacyl-tRNA synthetase disorders, but the genotype–phenotype association and natural history remain based on one patient [20].

For *IARS1*, *MARS1*, and *EIF2AK3*, the available literature predominantly consists of contextual multisystem disease reports, whereas qualifying recurrent PALF is uncommon and inconsistently documented. *IARS1*-related disease—classically described as growth retardation with impaired intellectual development, hypotonia, and hepatopathy—usually presents as a multisystem disorder, although infection-associated RALF can occur [6]. *MARS1* deficiency is distinguished by interstitial lung disease, which may accompany or precede hepatic involvement. These phenotypic clues should prompt genomic testing and careful respiratory and neurological assessment before LTx [6]. *EIF2AK3*-related Wolcott–Rallison syndrome results from dysfunction of the protein kinase RNA-like endoplasmic reticulum kinase (PERK) stress pathway. Permanent neonatal or early-infancy diabetes is the key diagnostic clue; recurrent hepatitis or liver failure may precede recognition of epiphyseal dysplasia. Acute episodes are often triggered by intercurrent illness and remain a major cause of death [21]. Meticulous diabetes management, avoidance of catabolism, and early treatment of infection are essential. Isolated LTx and simultaneous liver–pancreas transplantation have been reported, but evidence is confined to selected cases and must be weighed against multisystem disease and substantial transplant risk [22].

#### 3.2.4. Ion Homeostasis and Channel-Kinase Signaling

*TRPM7* encodes a ubiquitously expressed divalent-cation channel with an intracellular alpha-kinase domain. The channel conducts Mg^2+^, Zn^2+^, and Ca^2+^, and its activity is influenced by intracellular magnesium and Mg-nucleotide concentrations. Although these properties may be relevant to infection- or stress-associated liver injury, direct evidence from hepatocyte models is not yet available.

Evidence linking *TRPM7* to recurrent PALF is limited to a single discovery study of five previously unsolved children from three unrelated families; the reported episodes met strict or probable recurrent PALF criteria. The children were enrolled in the Pediatric Acute Liver Failure Exome Sequencing study. Exome reanalysis identified rare biallelic *TRPM7* variants after established PALF-associated genes had been excluded. Potentially damaging biallelic variants were enriched in the PALF cohort compared with a pediatric control cohort, and all affected children carried at least one variant involving the alpha-kinase region; one child additionally carried a channel-domain variant. This domain distribution raises the possibility that impaired kinase function is particularly relevant to the hepatic phenotype, but the cohort is too small to establish a domain-specific genotype-phenotype relationship.

The phenotype was dominated by early-onset, predominantly infection-triggered PALF with recurrence in most patients and rapid clinical deterioration; three of the five children died in early childhood. Routine metabolic investigations did not reveal a consistent diagnostic fingerprint. Marked hypomagnesemia was reported in only one child, who carried the channel-domain variant, whereas hypomagnesemia was not a shared feature of the remaining cases. Consequently, serum magnesium may be included in the biochemical assessment, but a normal value cannot exclude *TRPM7*-associated disease and isolated hypomagnesemia should not be regarded as a defining biomarker.

Functional support consisted mainly of proteomic analyses in patient-derived fibroblasts demonstrating reduced *TRPM7* protein abundance. These findings support an effect of the variants on protein stability or expression but do not establish how the variants cause liver failure. No validated biochemical marker, prospective surveillance protocol, targeted treatment, or reliable transplantation outcome estimate is available. *TRPM7* should therefore be considered in exome- or genome-based analysis of unexplained, particularly infection-triggered RALF after common metabolic, immune, infectious, and toxic causes have been assessed. Interpretation should integrate segregation, population frequency, domain location, computational evidence, and—where feasible—RNA, protein, or other functional studies. Current evidence remains very low certainty because it is based on five patients from one report [23].

#### 3.2.5. Disorders of Vesicular Trafficking

**Figure 3:**
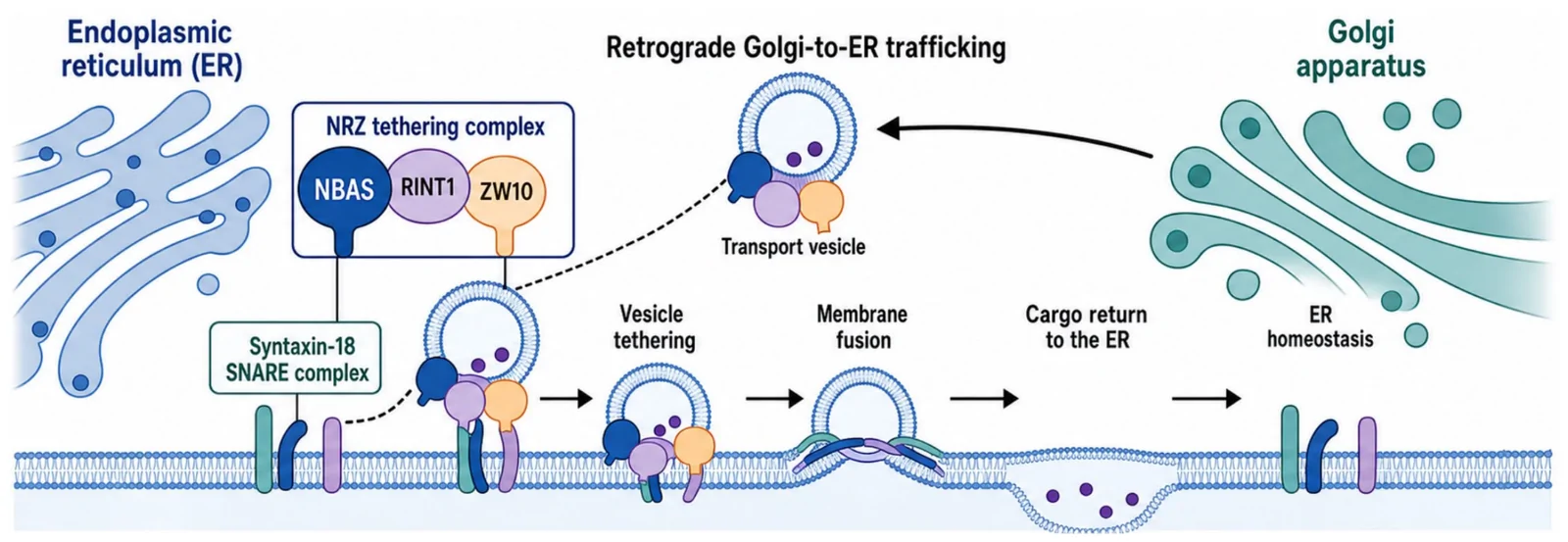
Role of the NBAS–RINT1–ZW10 tethering complex in retrograde Golgi-to-endoplasmic reticulum trafficking. The complex cooperates with the syntaxin-18 soluble N-ethylmaleimide-sensitive factor attachment protein receptor machinery in vesicle tethering and membrane fusion. ER, endoplasmic reticulum; NRZ, NBAS–RINT1–ZW10; SNARE, soluble N-ethylmaleimide-sensitive factor attachment protein receptor.

*NBAS*-, *RINT1*-, and *SCYL1*-associated diseases form the principal group of vesicular-trafficking disorders presenting with fever-associated RALF. All three are autosomal recessive and disrupt membrane transport between the endoplasmic reticulum (ER) and Golgi apparatus, but their natural histories are not interchangeable. NBAS and RINT1 are components of the NBAS–RINT1–ZW10 (NRZ) tethering complex required for docking and fusion of Golgi-derived coat protein complex I vesicles with the ER membrane (Figure 3). SCYL1 acts predominantly as a coat protein complex I-associated scaffolding protein at the cis-Golgi. Impaired retrograde transport compromises membrane recycling, protein retrieval, autophagy, and ER homeostasis; disturbed anterograde trafficking may additionally impair secretion of proteins in tissues with high secretory demand. ER stress and activation of cell-death pathways have been observed experimentally and may contribute to fever-triggered crises [6].

The clinical distinction is important. *NBAS*- and *RINT1*-associated crises are usually hepatocellular, with abrupt and sometimes high elevation of ALT activity, rapid deterioration of coagulation, and relatively limited cholestasis. Liver tests may normalize between attacks, and crisis frequency often decreases with age, although this age-related attenuation is supported much more strongly for *NBAS* than for *RINT1*. *SCYL1* deficiency more often produces recurrent low-gamma-glutamyl transferase (GGT) cholestasis and may progress to chronic structural liver disease despite fewer acute crises. None of the three disorders has a specific circulating biomarker. Their diagnosis therefore depends on recognition of the fever-associated pattern, systematic assessment of extrahepatic clues, and early genomic testing [6].

##### *NBAS*-Associated Disease

The evidence for *NBAS*-associated RALF includes strict cases together with partially overlapping disease cohorts that also contain single-episode ALF, recurrent liver injury, and nonhepatic phenotypes. NBAS is a component of the NBAS–RINT1–ZW10 tethering complex, which cooperates with the syntaxin-18 machinery in retrograde Golgi-to-endoplasmic reticulum transport (Figure 3); NBAS also participates in nonsense-mediated messenger RNA decay [24,25]. Patient-derived cells show impaired vesicle tethering, reduced stability of interacting proteins, endoplasmic-reticulum stress, and temperature-sensitive dysfunction. Experimental exposure to febrile temperature increases reactive oxygen species and apoptosis, providing a plausible explanation for fever-triggered liver crises [26,27].

The first hepatic episode usually occurs in infancy or early childhood. ALT activity may rise rapidly, with variable hypoglycemia, hyperammonemia, hyperbilirubinemia, and coagulopathy; biochemical recovery between attacks can be complete. In the international cohort of 230 individuals, 63.4% had liver involvement, 41.3% experienced at least one episode meeting ALF criteria, native-liver survival was 83.9%, and the latest documented episode occurred at 24 years [28]. Crisis frequency usually decreases with age, but persistent ALT elevation and progressive fibrosis have been reported [28,29].

The acute hepatic phenotype is characterized by a disproportionately rapid rise in ALT activities, frequently into the thousands, followed by impaired coagulation. Vomiting, reduced intake, hypoglycemia, hyperammonemia, jaundice, hepatomegaly, and encephalopathy occur variably. Fever or infection is the dominant trigger, and temperature itself may aggravate dysfunction of the thermolabile trafficking machinery; fasting, surgery, vaccination-associated fever, or other catabolic stress can also precipitate attacks. Histology during or after a crisis is nonspecific and may show hepatocellular injury, steatosis, inflammation, or fibrosis. A nondiagnostic biopsy therefore does not exclude *NBAS*-associated disease, and biopsy should not delay molecular testing.

Extrahepatic findings may include proportionate short stature, osteopenia, delayed bone age, vertebral abnormalities, fractures, optic atrophy, retinal or visual abnormalities, Pelger–Huët anomaly, characteristic facial features, hypotonia, developmental disability, and occasional endocrine disease. Hypogammaglobulinemia and quantitative or functional natural-killer- and B-cell abnormalities may increase susceptibility to infection and can simultaneously increase the probability of a fever-triggered hepatic crisis [30,31]. Genotype–phenotype associations are useful but not absolute: Sec39-domain variants are enriched in liver-predominant infantile liver failure syndrome type 2; C-terminal variants, including the Yakut founder variant, more often cause short stature, optic atrophy, and Pelger–Huët anomaly; and variants affecting the N-terminal β-propeller region are more often associated with a severe combined phenotype [32,33].

Management is centered on a written emergency protocol initiated at the onset of fever, not only after biochemical liver injury becomes apparent. It should include prompt antipyretic treatment, avoidance of fasting, carbohydrate-rich oral intake when tolerated, early intravenous glucose when intake is inadequate, and prompt investigation and treatment of infection according to clinical findings. The benefit of N-acetylcysteine (NAC), corticosteroids, or other pharmacological agents has not been established specifically for *NBAS* deficiency. LTx eliminates further liver-derived crises and is reasonable after life-threatening or increasingly severe episodes, failure to recover synthetic function, progressive fibrosis or portal hypertension, or recurrent crises that remain unacceptable despite optimal emergency management. It does not correct growth, skeletal, visual, neurological, or immunological manifestations; these must be characterized before listing and monitored after transplantation [28,34,35].

##### *RINT1*-Associated Infantile Liver Failure Syndrome Type 3

Evidence for *RINT1*-associated RALF is derived from small strict or probable RALF series with limited transplantation follow-up. Biallelic *RINT1* variants cause infantile liver failure syndrome type 3 (ILFS3). *RINT1* is a component of the NRZ complex and also interacts with proteins regulating autophagy, explaining both the clinical overlap with *NBAS*-associated disease and the broader cellular phenotype. Patient fibroblasts have shown reduced RINT1 abundance, secondary reduction of NBAS and ZW10, abnormal Golgi morphology, and impaired autophagic flux. The small number of patients and absence of individuals with confirmed biallelic complete loss-of-function variants suggest that substantial residual *RINT1* function may be required for survival [6,36].

The first liver crisis generally occurs in infancy or early childhood and is precipitated by fever or infection. Reported attacks include vomiting, reduced intake, marked ALT activity elevation, hypoglycemia, hyperammonemia, coagulopathy, jaundice, and encephalopathy. Recovery may be complete, but some patients retain mildly abnormal ALT levels between episodes. Liver biopsies have shown nonspecific steatosis, fibrosis, hepatocellular injury, or increased Kupffer cells. Severe outcomes, including death during ALF, have also occurred. Conversely, individual patients have remained clinically stable for prolonged periods, emphasizing that the presently documented cases cannot support reliable estimates of age-specific mortality, recurrence, or transplant-free survival [6,36].

The extrahepatic phenotype is heterogeneous. Short stature, delayed bone age, vertebral and pelvic abnormalities, kyphosis, osteopenia, fractures, hypotonia, spasticity, ataxia, optic-nerve abnormalities, and developmental disability have been reported. More recent observations have expanded the phenotype to include neutropenia, whereas prominent skeletal or neurological disease may be absent. These findings argue against excluding RINT1 deficiency solely because a child lacks the originally described skeletal phenotype.

No disease-specific biochemical marker is available. RINT1 deficiency should therefore be included in rapid sequencing panels or exome/genome analysis when fever-associated RALF remains unexplained, irrespective of whether skeletal or neurological abnormalities are already apparent. Acute treatment follows the same principles as for NBAS deficiency: rapid control of fever, avoidance of fasting, early carbohydrate delivery, intravenous glucose when necessary, treatment of infection. A written emergency regimen is appropriate, but no disease-specific drug therapy has been validated.

Evidence for LTx in ILFS3 is confined to very few cases. One of the originally reported patients underwent transplantation at eight years of age, but the literature is insufficient to determine whether outcomes differ from those of other RALF disorders or whether transplantation modifies long-term extrahepatic progression [36]. Referral should be based on the severity, recurrence, and reversibility of hepatic crises; evidence of chronic structural liver injury; quality-of-life burden; and neurological, skeletal, and immunological assessment. LTx may prevent liver-derived decompensation but cannot be assumed to correct the systemic consequences of *RINT1* deficiency.

##### *SCYL1*-Associated CALFAN Syndrome

The *SCYL1* literature includes recurrent PALF, recurrent cholestatic injury, and progressive acute-on-chronic liver disease; these clinically distinct phenotypes are considered separately. Biallelic *SCYL1* variants cause CALFAN syndrome, named for low-gamma-glutamyl transferase (GGT) cholestasis, ALF triggered by febrile infections, and neurodegeneration. SCYL1 is a catalytically inactive kinase-like protein involved in coat protein complex I-mediated retrograde trafficking, Golgi morphology, and cellular stress responses. Loss of *SCYL1* disrupts ER–Golgi homeostasis and may impair autophagy and protein secretion. Unlike the predominantly hepatocellular crises of NBAS and RINT1 deficiencies, *SCYL1*-associated liver disease frequently includes conjugated hyperbilirubinemia with an inappropriately low or normal gamma-glutamyl transferase activity [6,37].

Hepatic manifestations usually begin in infancy or early childhood with fever-associated episodes of vomiting, jaundice, hepatomegaly, elevated ALT, coagulopathy, and occasionally encephalopathy. Some children recover nearly normal synthetic function between episodes, and acute attacks may become less frequent with age. This apparent attenuation does not necessarily indicate resolution: persistent cholestasis, splenomegaly, fibrosis, portal hypertension, cirrhosis, or recurrent jaundice may develop. Liver histology may show cholestasis, steatosis, fibrosis, or cirrhotic remodeling, but findings are not specific [6,37].

Neurological disease may appear after the hepatic presentation and progress independently of biochemical liver recovery. Peripheral neuropathy, lower-limb weakness, reduced or absent reflexes, tremor, cerebellar ataxia, dysarthria, developmental disability, optic atrophy, and cerebellar atrophy have been reported. Skeletal abnormalities, short stature, and rare episodes of respiratory insufficiency further broaden the phenotype. Regular neurological examination, developmental assessment, ophthalmological review, and—when indicated—nerve-conduction studies and brain imaging are therefore essential, including in children whose liver crises have diminished.

Acute management requires immediate anti-catabolic support, treatment of the febrile trigger, and standard PALF care. A written fever protocol is appropriate, but no pharmacological treatment has been shown to correct the trafficking defect. The decision about LTx is particularly complex because acute crises may attenuate while chronic cholestatic liver disease and neurodegeneration continue. LTx may be appropriate for non-recovering ALF, recurrent life-threatening decompensation, progressive cirrhosis, portal hypertension, or deterioration of synthetic function. Successful hepatic outcome with a functioning graft at three years has been reported after transplantation in early adulthood, but this single experience cannot define neurological benefit or long-term prognosis [38]. LTx cannot be expected to arrest established peripheral or central neurodegeneration; neurological trajectory, mobility, respiratory function, nutritional status, and rehabilitation potential must therefore be assessed explicitly before listing and followed after transplantation.

### 3.3. Diagnostic Approach to RALF

The diagnostic clue is the recurrent pattern rather than a single laboratory value: rapidly evolving hepatocellular injury or cholestatic dysfunction during infection or catabolic stress, impaired hepatic synthetic function, and substantial recovery between attacks. RALF should first be distinguished from repeated acute decompensation of unrecognized chronic liver disease and from recurrent ALT activity elevation without coagulopathy. Previous medical records should be reconstructed whenever possible, including age at each episode, trigger, maximum INR, aminotransferase and ammonia concentrations, bilirubin pattern, glucose and lactate values, time to recovery, treatments, and whether liver tests normalized between attacks. Hyperammonemia, lactic acidosis, hypoglycemia, neonatal diabetes, interstitial lung disease, short stature, skeletal abnormalities, optic atrophy, neuropathy, cytopenia, abnormal neutrophil morphology, or immune dysfunction can direct the initial differential diagnosis but may be absent at the first crisis.

During an acute presentation, standard PALF evaluation and stabilization must proceed in parallel with metabolic and genetic investigations. Immediate tests should include prothrombin time and serial INR after parenteral vitamin K when appropriate, alanine and aspartate aminotransferases, gamma-glutamyl transferase, alkaline phosphatase, total and conjugated bilirubin, albumin, glucose, ammonia, lactate, blood gas, electrolytes, phosphate, renal function parameters, creatine kinase, full blood count, and ferritin, triglycerides, and fibrinogen when hemophagocytic lymphohistiocytosis (HLH) is suspected. Trends are more informative than isolated values: a rapidly increasing INR, recurrent hypoglycemia, rising ammonia or lactate, and failure of synthetic recovery should accelerate transfer to a specialist pediatric liver and transplant center. Infection, autoimmune hepatitis, drug or toxin exposure—including a measured paracetamol concentration when relevant—Wilson disease where age-appropriate, gestational alloimmune liver disease in neonates, mitochondrial hepatopathy, fatty-acid oxidation defects, and other metabolic causes should be investigated according to age and presentation [1,39].

Critical metabolic samples should be obtained as early as possible, preferably before prolonged protein restriction, dialysis, transfusion, or large glucose loads alter the biochemical profile, but sampling must never delay treatment. The minimum set should include plasma amino acids, acylcarnitine profile, total and free carnitine, urinary organic acids, urinary orotic acid, urinary amino acids when HHH syndrome is considered, blood or plasma ketones, and stored plasma, urine, and EDTA blood for repeat or specialized studies. In hyperammonemia, low or low-normal citrulline with increased urinary orotic acid supports OTC deficiency, whereas hyperornithinemia and urinary homocitrulline suggest HHH syndrome. Results may be blunted by reduced protein intake, and a normal single sample does not exclude either disorder.

Lactic acidosis accompanied by hypoglycemia should prompt evaluation for *TRMU* and *DLD* deficiencies and other mitochondrial hepatopathies. Lactate should be interpreted with sampling technique, perfusion, seizures, and hepatic clearance in mind; neither lactate nor the lactate-to-pyruvate ratio is specific for mitochondrial disease during PALF. Plasma amino acids, acylcarnitines, and urinary organic acids may show secondary or disease-related abnormalities in *DLD* deficiency but can be normal between crises and occasionally during decompensation. In suspected *TRMU* deficiency, persistent infantile lactic acidosis combined with liver failure is an important clue, but molecular confirmation remains essential.

Because transplant decisions may be time-critical, genomic testing should be initiated during the first unexplained severe episode and pursued in parallel with clinical stabilization [40]. Rapid trio exome or genome sequencing is preferable when the phenotype is not diagnostic, particularly in neonates and critically ill infants. A targeted liver-failure panel is reasonable only when it offers a shorter validated turnaround time and comprehensive coverage of urea-cycle, mitochondrial, protein-translation and cellular-stress, ion-homeostasis and channel-kinase, and vesicular-trafficking genes. Analysis must include copy-number variants and should not be restricted to previously reported variants. Negative exome sequencing does not end the investigation: poorly covered exons, structural variants, mitochondrial DNA, complex rearrangements, splice-altering variants, and deep intronic variants may require genome sequencing, RNA studies, quantitative protein analysis, enzyme assays, or other functional investigations [41].

Genetic results require phenotype-aware interpretation. A variant of uncertain significance should not be used alone to justify or withhold LTx, whereas a plausible molecular finding may need rapid segregation testing and functional confirmation. Conversely, treatment should not wait for sequencing when a recognizable and immediately treatable biochemical pattern is present. Molecular confirmation is particularly important when considering LTx in a multisystem disorder because replacement of the liver may correct hepatic metabolism or prevent liver-derived crises without modifying neurological, muscular, skeletal, immunological, pulmonary, or endocrine disease.

After a molecular diagnosis, genetic counselling should address the mode of inheritance, recurrence risk, segregation testing in the parents, targeted testing of at-risk relatives, and reproductive options. For autosomal-recessive disorders, when both parents are confirmed heterozygous carriers, each pregnancy carries a 25% probability of an affected child, a 50% probability of a heterozygous carrier, and a 25% probability of a child who has inherited neither familial variant. In X-linked OTC deficiency, risk assessment should take into account the genetic status of the mother, the sex of the offspring, and the variable clinical expression in heterozygous females. A variant that appears to have arisen de novo should be confirmed, and parental testing should be undertaken when feasible; even when the variant is absent from parental blood samples, a low residual recurrence risk remains because of possible germline mosaicism. Targeted testing of asymptomatic siblings and other at-risk relatives should be offered when the result could alter surveillance, emergency planning, preventive measures, or treatment. Once a familial pathogenic or likely pathogenic variant has been identified, prenatal diagnosis and preimplantation genetic testing may be discussed as part of non-directive reproductive counselling. A variant of uncertain significance should not be used for predictive testing or reproductive decision-making unless additional evidence leads to its reclassification.

The inherited differential diagnosis should be prioritized according to the dominant biochemical and clinical pattern. Marked hyperammonemia suggests OTC deficiency or HHH syndrome, particularly when infection, increased protein intake, catabolism, or fasting precipitates the episode. Infantile lactic acidosis, hypoglycemia, and liver failure suggest TRMU deficiency; recurrent vomiting, hypoglycemia, lactic acidosis, and hepatocellular injury from infancy to adulthood are compatible with hepatic *DLD* deficiency. Growth failure, microcytic anaemia, hypoalbuminemia, hypotonia, or developmental disability can indicate *LARS1* deficiency, while a broader neurodevelopmental phenotype suggests *IARS1*-related disease and interstitial lung disease is an important clue to MARS1 deficiency. Permanent neonatal or early-childhood diabetes strongly suggests *EIF2AK3*-related Wolcott–Rallison syndrome, even before epiphyseal dysplasia becomes evident [6]. Microcephaly and developmental delay accompanying fever-triggered episodes may indicate AARS1 deficiency, although this association is currently based on one patient [20]. *TRPM7* defect should be considered in severe unexplained infection-associated RALF, including cases without a recognizable metabolic fingerprint; serum magnesium may be informative but a normal value does not exclude the disorder [23].

A predominantly hepatocellular crisis with fever, very high aminotransferases, limited cholestasis, and near-complete interval recovery should raise NBAS or RINT1 deficiency. Short stature, optic atrophy, Pelger–Huët anomaly, fractures, hypogammaglobulinemia, or natural-killer- and B-cell abnormalities favour *NBAS*-associated disease; vertebral or pelvic abnormalities, spasticity, ataxia, or neutropenia may support RINT1 deficiency. Recurrent conjugated hyperbilirubinemia with low or normal gamma-glutamyl transferase, followed by peripheral neuropathy or cerebellar signs, is more characteristic of *SCYL1*-associated CALFAN syndrome. Absence of extrahepatic findings early in life does not exclude any of these diagnoses.

Evaluation must continue after apparent recovery. Interval assessment should document whether INR, bilirubin, aminotransferases, ammonia, lactate, and glucose normalize; screen for fibrosis, portal hypertension, or splenomegaly; and reassess growth, vision, neurological development, muscle, heart, lungs, skeleton, blood count, and immune function according to the suspected disorder. Liver biopsy is reserved for questions that cannot be resolved noninvasively, such as unexplained chronic injury or competing diagnoses; its findings in genetic RALF are usually nonspecific and it should not postpone genomic testing. A staged diagnostic approach should be individualized and should combine emergency stabilization, first-line metabolic testing, infectious and toxicology assessment, and early genomic diagnostics when the first-line evaluation is unrevealing [1,39,40,41,42,43].

Figure 2 and Table 1 can be used as a bedside sequence rather than as a linear checklist. The immediate priority is stabilization: secure airway and circulation when needed, correct hypoglycaemia, avoid fasting, treat suspected infection, and involve a pediatric liver or transplant centre early. Critical metabolic samples should be obtained as soon as feasible, especially ammonia, lactate, blood gas, glucose, plasma amino acids, acylcarnitines, urinary organic acids, and stored DNA; sampling should not delay anti-catabolic treatment or ammonia-lowering therapy when clinically indicated. If the first severe episode remains unexplained after initial biochemical assessment, rapid trio exome or genome sequencing should be initiated during the acute admission rather than deferred until a second crisis.

A comparative summary of key inherited disorders and a staged diagnostic approach are provided in Table 1 and Table 2.

### 3.4. Management of Acute Liver Crises

**Figure 4:**
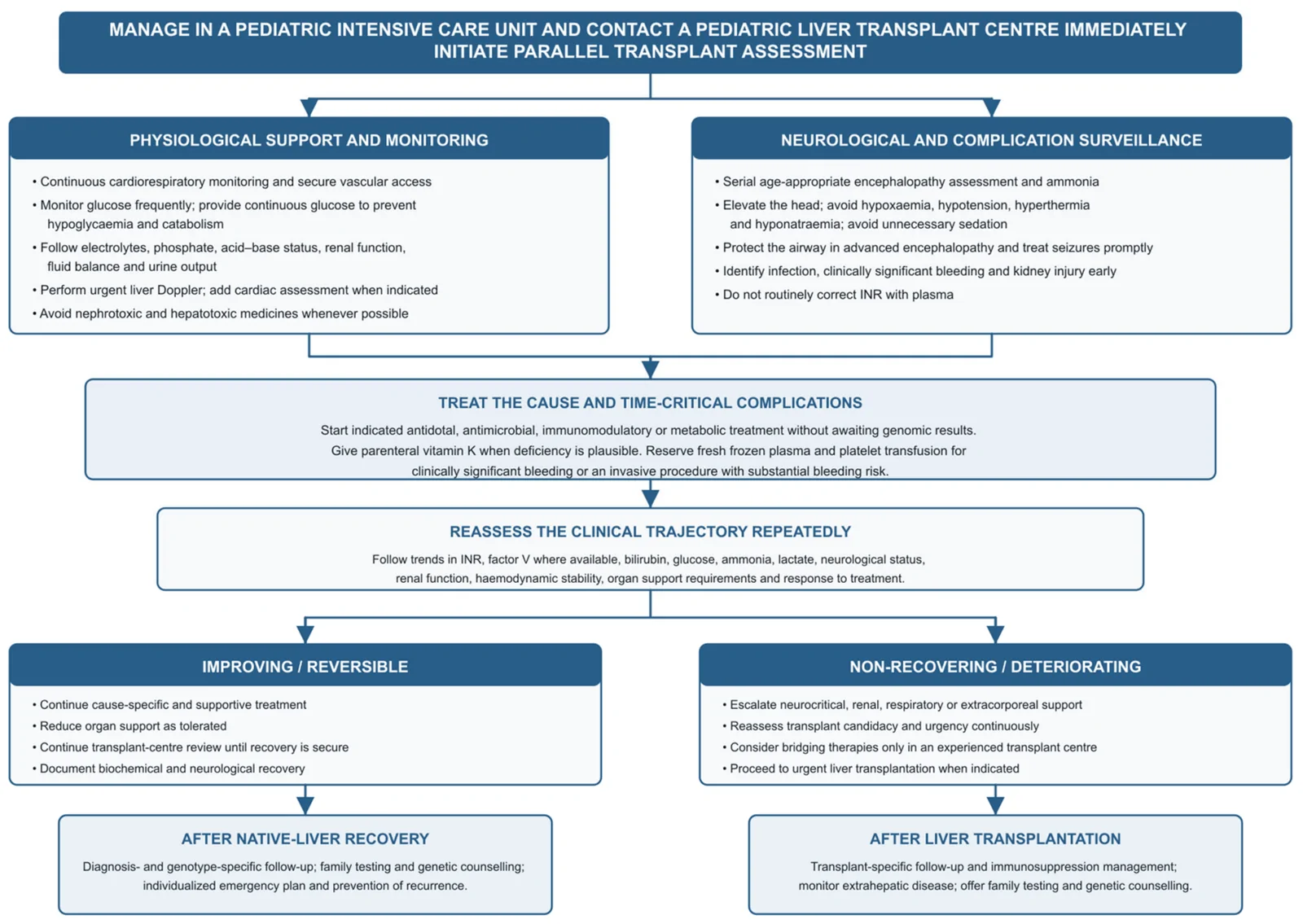
Author-proposed management pathway for pediatric acute liver failure. Intensive supportive care, cause-specific treatment and surveillance for neurological, renal, infectious and haemostatic complications proceed in parallel, with repeated reassessment of reversibility and transplant candidacy.

Stabilization and disease-specific treatment must proceed simultaneously, preferably in a pediatric intensive-care setting with early involvement of a specialist liver and transplant center. Management includes frequent assessment of glucose, ammonia, lactate, acid–base status, INR, bilirubin, electrolytes, phosphate, renal function, fluid balance, and neurological state; immediate treatment of infection; avoidance of fasting; and prompt provision of enteral or intravenous non-protein energy (Figure 4). Airway protection, management of cerebral edema and seizures, renal support, and correction of clinically relevant electrolyte or acid–base disturbances follow standard PALF principles. Vitamin K should be given when deficiency is possible, but prophylactic plasma solely to normalize INR should generally be avoided in the absence of bleeding or an invasive procedure because it obscures the trajectory of hepatic synthetic function [1,43].

Disease-specific measures should be started on clinical and biochemical suspicion. Severe hyperammonemia requires immediate nitrogen-scavenging therapy and early consideration of extracorporeal ammonia removal, particularly when ammonia continues to rise, neurological status deteriorates, or medical treatment does not produce a rapid decline. Urea-cycle decompensation requires temporary protein interruption, high-energy glucose with or without lipids, and arginine or citrulline according to the defect; prolonged complete protein withdrawal should be avoided once metabolic control is achieved. Suspected *TRMU* deficiency warrants early cysteine-source supplementation. *DLD*-related crises require aggressive glucose delivery and avoidance of catabolism; riboflavin is commonly used despite uncertain efficacy. Fever-triggered *NBAS*, *RINT1*, *SCYL1*, and aminoacyl-transfer RNA synthetase disorders require prompt temperature control, hydration, carbohydrate provision, and treatment of the precipitating infection [6,10,12,15,17].

A written emergency protocol is particularly important in fever-triggered translation and trafficking disorders. It should specify when oral carbohydrate intake is insufficient, when intravenous glucose should begin, which laboratory tests must be repeated, and when the child should be transferred to a specialist center. Fluid composition and glucose delivery must be individualized to age, nutritional status, cardiac and renal function, and severity of liver failure. Early transplant-center referral is required when INR continues to rise despite vitamin K, encephalopathy develops, ammonia or lactate cannot be controlled, hypoglycemia recurs, multiorgan dysfunction emerges, or spontaneous recovery is uncertain. Referral is not equivalent to immediate listing: it preserves access to urgent LTx while diagnostic and disease-specific treatment continue.

NAC has three distinct contexts in RALF. First, it is established treatment for paracetamol poisoning and should be administered promptly when paracetamol toxicity is confirmed or cannot initially be excluded. Second, routine NAC for all non-paracetamol PALF is not supported by pediatric randomized evidence and should not replace etiological treatment or timely transplant assessment [43,44]. Third, disease-specific biological rationales exist. In *TRMU* deficiency, NAC functions as a cysteine source and is commonly combined with L-cysteine; retrospective data associate cysteine-source supplementation with improved survival, although formulation, dose, route, and duration remain unstandardized [15,45,46]. In *NBAS*-deficient cells, NAC reduced fever-associated reactive oxygen species accumulation and apoptosis, and a small retrospective clinical series suggested biochemical benefit during febrile episodes [27].

The *NBAS* evidence remains hypothesis-generating: no randomized trial, validated dose, or standardized treatment threshold exists. NAC may be considered as an adjunct in selected *NBAS*-associated crises in specialist-led care, but it must not delay glucose delivery, infection treatment, intensive monitoring, genomic investigation, or transplant assessment. Outside paracetamol toxicity and suspected or confirmed *TRMU* deficiency, its use should be individualized rather than presented as routine therapy for genetic RALF.

### 3.5. Long-Term Surveillance and LTx

Follow-up after recovery should be individualized according to the molecular diagnosis, hepatic course, and extrahepatic involvement. An early post-discharge review should assess the trajectory of INR, bilirubin, aminotransferases, glucose, and—when indicated by the phenotype—ammonia or lactate, and should reassess growth, nutrition, neurological status, and development after critical illness. Subsequent visits should document recurrent episodes and treatment burden. Evaluation for fibrosis or portal hypertension, using platelet count, spleen assessment, Doppler ultrasonography, and non-invasive fibrosis testing, should be reserved for children with persistent biochemical or clinical abnormalities or a disorder associated with chronic liver disease. Renal, cardiac, respiratory, ophthalmological, skeletal, immunological, haematological, and endocrine surveillance should be tailored to the underlying disorder. Children who undergo liver transplantation require a dedicated transplant-centre follow-up programme. Families of children at risk of recurrent fever- or catabolism-triggered decompensation should receive an individualized emergency plan covering fever, fasting, surgery, and prolonged vomiting [6,29].

The role of LTx in RALF extends beyond rescue of a single non-recovering episode. It may replace a liver-specific enzyme defect, prevent recurrent liver-derived crises, remove the risk of sudden fatal decompensation, and reduce dietary and emergency-treatment burden. However, it exposes a child who may recover spontaneously to operative mortality, lifelong immunosuppression, infection, renal toxicity, and graft complications. It also cannot reverse established neurological injury and may not correct a defect expressed in brain, muscle, skeleton, immune system, pancreas, or other tissues. The number of previous episodes alone is therefore an inadequate indication. Decisions should integrate severity and reversibility of recent crises, maximum ammonia and INR, neurological injury, interval hepatic function, fibrosis or portal hypertension, genotype and expected natural history, effectiveness of disease-specific treatment, extrahepatic prognosis, quality of life, family capacity to implement emergency treatment, and access to urgent specialist care.

In OTC deficiency and HHH syndrome, LTx restores effective hepatic ureagenesis and can prevent further hyperammonemic crises, allowing substantial relaxation of protein restriction and nitrogen-scavenger therapy. It should be considered before irreversible neurological injury in severe neonatal-onset OTC deficiency, recurrent medically uncontrolled hyperammonemia, poor metabolic control, or unacceptable neurological risk; established brain injury will not be reversed [10,13]. In *NBAS*-associated disease, LTx prevents further hepatic crises but does not correct growth, skeletal, ophthalmological, or immunological disease [34,35]. Experience in RINT1 deficiency is too limited for a disease-specific threshold. In SCYL1 deficiency, progressive cholestasis, cirrhosis, portal hypertension, or non-recovering ALF may justify transplantation, but neurological progression may continue after successful hepatic replacement [37,38].

TRMU deficiency requires a deliberately conservative approach because ALF is commonly reversible and largely restricted to infancy. Among 11 reported recipients, LTx was not associated with improved overall survival; transplantation should be reserved for irreversible deterioration despite optimal supportive and cysteine-source treatment, with attention to genotype and extrahepatic disease [15]. Evidence in hepatic *DLD* deficiency is even weaker: the only transplanted patient in the largest review died despite graft engraftment, so LTx cannot be assumed to prevent systemic metabolic failure [17,18]. For *LARS1*, *IARS1*, *MARS1*, and *EIF2AK3*-related disease, published experience is sparse and multisystem prognosis is central; isolated or combined liver–pancreas transplantation in Wolcott–Rallison syndrome remains exceptional [19,21,22].

Transplant evaluation should begin before an emergency forces a binary decision. During a stable interval, the team can define neurological and developmental status, cardiac and respiratory risk, renal function, nutritional reserve, infection and immunological status, vascular anatomy, and the likelihood that native-liver recovery will persist. During an acute crisis, serial trajectory is more informative than a single INR or aminotransferase value. Listing should remain dynamic: improvement with metabolic treatment may justify delisting or continued observation, whereas worsening synthetic failure, uncontrollable hyperammonemia, cerebral injury, or multiorgan deterioration may require urgent LTx. The disease-specific role and limitations of transplantation should be interpreted in the context of the underlying molecular diagnosis, recovery between crises, and extrahepatic prognosis.

Published transplant experience must be interpreted at two complementary levels. In the most recent individual-participant analysis of genetically confirmed RALF caused by NBAS, RINT1, SCYL1, or LARS1 deficiency, 18 recipients underwent LTx (11 with NBAS deficiency, five with SCYL1 deficiency, one with RINT1 deficiency, and one with LARS1 deficiency). Median age at LTx was 2.3 years; no patient had recurrent ALF after LTx, although two post-transplant deaths were reported and follow-up was heterogeneous [47]. An additional Chinese case series described three children with NBAS deficiency who underwent LTx; two survived, whereas one died from severe infection within one month after transplantation [48]. Larger disease-specific cohorts provide additional but partly overlapping aggregate data: 16 recipients with *NBAS*-associated disease had a median post-transplant follow-up of 5 years, whereas 11 recipients with TRMU deficiency underwent LTx at a median age of 4 months without a demonstrated overall-survival benefit [15,28]. Because many reports do not provide complete recipient-level follow-up and duplicate patients across publications, individually traceable cases and aggregate cohorts should be considered separately and must not be summed (Supplementary Table S1).

The role of liver transplantation differs substantially among the genetic disorders associated with recurrent acute liver failure. In severe urea-cycle disorders, particularly OTC deficiency, transplantation is an established therapeutic option that can restore hepatic ureagenesis and prevent further hyperammonaemic crises. It should be considered before recurrent metabolic decompensation causes irreversible neurological injury. Experience in HHH syndrome is more limited, and transplantation decisions should therefore be individualized. For *NBAS*-, *RINT1*-, *SCYL1*-, and *LARS1*-associated disease, the available evidence is derived mainly from case reports and small retrospective series. The absence of further hepatic crises after transplantation suggests that replacing the liver can control the hepatic component of these disorders, but these observations do not define optimal timing, demonstrate a survival benefit, or establish a role for pre-emptive transplantation. This is particularly important because the frequency of hepatic episodes may decrease with age, whereas neurological and other extrahepatic manifestations may persist or progress. TRMU deficiency requires a different approach because hepatic dysfunction is often transient and may respond to metabolic treatment; transplantation should generally be reserved for progressive or non-recovering liver failure despite appropriate therapy. In hepatic DLD deficiency, transplantation may control liver failure in carefully selected patients, but persistent or progressive systemic mitochondrial disease must be considered. For *IARS1*-, *MARS1*-, *AARS1*-, and *TRPM7*-associated disease, and most other multisystem aminoacyl-tRNA synthetase disorders, the evidence is insufficient to support disorder-specific transplantation recommendations. Across all conditions, successful transplantation in an individual patient demonstrates feasibility but does not by itself establish optimal timing or clinical benefit.

## 4. Discussion

This review identifies recurrent acute liver failure as a convergent clinical phenotype rather than a discrete disease category. The same sequence—an apparently healthy child, an intercurrent infection or catabolic stress, rapid deterioration of hepatic synthetic function, and partial or complete interval recovery—can result from disturbances of nitrogen disposal, mitochondrial translation or energy metabolism, cytosolic protein translation and cellular stress, ion homeostasis and channel-kinase signaling, or vesicular trafficking. The value of the five-group framework is therefore practical rather than taxonomic: it organizes early diagnostic reasoning while emphasizing that phenotypic overlap is substantial and that a normal first-line metabolic profile does not exclude a genetic cause.

### 4.1. Defining RALF as a Clinical Phenotype

An important limitation of the available literature is the inconsistent use of the term “recurrent acute liver failure.” Some reports describe repeated episodes that fulfil PALF coagulopathy criteria, with recovery of coagulation parameters between episodes. Others use the same term for recurrent aminotransferase elevation, jaundice, or metabolic decompensation without adequately documented liver failure. Although these presentations may occur within the same disorders, they should not be regarded as equivalent. Combining them may overestimate the occurrence of RALF and make it difficult to distinguish recurrent liver failure from recurrent liver injury or acute deterioration in a child with pre-existing fibrosis or cirrhosis. Distinguishing strict RALF from broader contextual evidence helps clarify these differences but also shows that episode-level clinical and biochemical data are available for relatively few published patients [1,2].

The clinical and biochemical state between episodes is equally important. Normalization of aminotransferase activity alone does not confirm complete hepatic recovery, whereas persistent minor biochemical abnormalities do not necessarily indicate chronic decompensated liver disease. Future reports should include serial INR values, interpreted in relation to vitamin K, plasma, and coagulation-factor administration; total and conjugated bilirubin; glucose; ammonia; lactate; the duration of each episode; and the time and extent of recovery. The presence or absence of clinical, imaging, elastographic, or—when clinically obtained—histological evidence of chronic liver disease should also be reported. More complete longitudinal documentation would help distinguish recurrent PALF from recurrent liver injury and acute-on-chronic liver disease and would allow more meaningful comparisons among genetic disorders.

### 4.2. Clinical Meaning of the Five-Group Framework

The five groups share a clinical phenotype of stress-dependent hepatocyte failure, but the upstream biology and diagnostic signals differ. Urea-cycle disorders are distinguished by hyperammonemia and characteristic amino-acid or urinary orotate patterns; mitochondrial disorders more often combine hypoglycemia, lactic acidosis, and multisystem energy failure; aminoacyl-tRNA synthetase and related stress-response disorders may be accompanied by growth, neurological, pulmonary, skeletal, or endocrine abnormalities; *TRPM7*-associated disease currently lacks a reproducible biochemical signature; and vesicular-trafficking disorders are suggested by fever dependence, rapid hepatocellular injury, and variable skeletal, neurological, hematological, or immune features. These clues should prioritize urgent testing, but none is sufficiently sensitive to function as a gatekeeper for genomic analysis.

The grouping also shows why fever or fasting can precipitate attacks across otherwise different disorders. Protein breakdown can worsen nitrogen disposal; reduced substrate availability and increased energy demand can destabilize mitochondrial disease; fever can impair temperature-sensitive intracellular trafficking; and infection may exacerbate disorders of protein synthesis or ion regulation. These observations support early prevention of catabolism, although evidence for specific emergency regimens remains largely observational.

### 4.3. New and Emerging Genetic Etiologies

*AARS1* and *TRPM7* illustrate how the etiological spectrum continues to expand. *AARS1*-associated RALF is supported by a single functionally characterized patient, whereas the *TRPM7* association is based on five children from three families with genetic enrichment and fibroblast proteomic evidence [20,23]. These studies are important because both disorders may lack a conventional metabolic fingerprint. At the same time, neither report establishes prevalence, penetrance, the full phenotypic spectrum, or transplant responsiveness. They should therefore be regarded as emerging gene–disease associations requiring replication and rigorous variant interpretation rather than as diagnoses inferred solely from a rare variant.

*TRPM7* is particularly relevant to the conceptual breadth of RALF because it links a divalent-cation channel to an intracellular alpha-kinase. The observation of marked hypomagnesemia in only one patient is hypothesis-generating, not evidence that systemic magnesium deficiency explains the hepatic phenotype. Serum magnesium should be measured because it is clinically actionable and may support interpretation in an individual case, but normal magnesium neither excludes *TRPM7*-associated disease nor weakens a well-supported molecular diagnosis. Studies in hepatocyte models are needed to clarify the contributions of cation transport, kinase activity, protein stability, and cellular stress responses [23].

The absence of *HSD3B7* from the five-group synthesis reflects the phenotype-based eligibility criteria rather than lack of clinical importance. HSD3B7 deficiency typically causes neonatal or childhood low-GGT cholestasis with fat soluble vitamin malabsorption and progressive fibrosis or cirrhosis; severe liver failure may occur, but the retrieved reports did not document the required pattern of at least two discrete PALF episodes with interval restoration of synthetic function. It should remain in the differential diagnosis of cholestatic liver failure, while inclusion in a strict RALF synthesis would blur the boundary between recurrent PALF and progressive genetic cholestatic disease.

### 4.4. Implications for Diagnostic Strategy

The timing of diagnostic evaluation is particularly important. An inherited cause should be considered during the first unexplained episode of severe acute liver dysfunction rather than only after recurrence. Stabilization and treatment of immediately reversible causes must take priority, but diagnostic sampling should begin at the same time whenever feasible. Critical metabolic samples should be obtained early, preferably before dialysis, transfusion, prolonged protein restriction, or other interventions that may alter the results. Toxicological, infectious, immune, and metabolic investigations should be selected according to the child’s age, clinical presentation, biochemical pattern, and potential exposures. When a metabolic disorder is suspected, appropriate anti-catabolic and disease-specific treatment should not be delayed while awaiting diagnostic confirmation. Rapid trio exome or genome sequencing should be considered when the initial clinical and biochemical evaluation does not identify a cause, when the course is unusually severe or recurrent, when liver transplantation is being evaluated, or when extrahepatic findings suggest a multisystem disorder. Genomic testing has provided a substantial diagnostic yield in cohorts of children with indeterminate PALF; however, current evidence demonstrates improved diagnostic identification rather than improved survival or other clinical outcomes. Its early use should therefore be presented as an evidence-informed clinical strategy rather than a proven outcome-improving intervention [4,5].

A molecular finding is most informative when it is consistent with the clinical presentation, biochemical findings, and expected mode of inheritance. Pathogenic or likely pathogenic variants in a gene with an established disease association may guide acute treatment, surveillance, testing of relatives, and assessment of whether isolated liver transplantation is appropriate. These decisions should not be based on variant classification alone but on the combined molecular and clinical evidence. A variant of uncertain significance should not be used by itself to support or exclude listing for liver transplantation or to guide predictive testing and reproductive decisions. Evaluation may include segregation analysis, reassessment of the phenotype, review of population frequency and published cases, and—when appropriate—RNA, protein, or disease-relevant functional studies. Computational predictions may provide supporting evidence but should not be treated as independently diagnostic. Particular caution is required for associations supported by only a small number of patients, including the currently limited evidence linking *AARS1* and *TRPM7* to recurrent acute liver failure.

### 4.5. Therapeutic Interpretation and Liver Transplantation

The available literature supports several general principles of acute management: prompt treatment of hypoglycaemia, prevention of fasting and catabolism, early recognition and treatment of suspected infection, and timely involvement of metabolic, hepatology, intensive-care, and transplant specialists. Evidence supporting disorder-specific treatment is less consistent. Established therapies for urea-cycle disorders, including nitrogen-scavenging treatment and extracorporeal ammonia removal when indicated, are supported by broader clinical experience than most interventions proposed for other genetic causes of RALF. In TRMU deficiency, early L-cysteine or N-acetylcysteine supplementation may improve the course of liver disease, but the evidence remains based on small observational series. For *NBAS*-, *RINT1*-, and *SCYL1*-associated disease, management is primarily supportive and includes prevention of catabolism, prompt treatment of fever and infection, and early provision of carbohydrate during intercurrent illness [6,10,15,16,45,46]. Improvement following treatment during an episode that may resolve spontaneously cannot by itself establish efficacy. Reports should specify when treatment was started, the preparation, route and dose, accompanying supportive measures, the biochemical and clinical course, adverse effects, and the frequency and severity of subsequent episodes.

The role of LTx must be considered separately for each disorder. In selected urea-cycle disorders, transplantation can restore hepatic ureagenesis and prevent further hyperammonaemic decompensation, but it cannot reverse neurological injury that has already occurred. In *NBAS*-, *RINT1*-, *SCYL1*-, and related disorders, reported recipients generally did not experience further hepatic crises, suggesting that transplantation can control the liver component of the disease. The available reports do not, however, establish optimal timing or a survival advantage, and neurological or other extrahepatic manifestations may persist or progress. The potential benefit is more uncertain when hepatic recovery is common, as in many children with TRMU deficiency, or when systemic disease is likely to determine long-term outcome. Assessment should therefore consider whether liver disease is the main threat to survival and long-term health, whether the acute episode remains potentially reversible, and whether extrahepatic disease is compatible with a meaningful benefit from transplantation. Available studies are too small, heterogeneous, and vulnerable to selective reporting to permit reliable comparisons of transplant-free survival, graft survival, recurrence, or extrahepatic outcomes among genetic disorders [6,10,13,15,16,17,18,44,45,46].

Future reports and registries should provide the molecular diagnosis, number and severity of episodes before transplantation, evidence of chronic liver disease, indication and urgency of transplantation, age at transplantation, donor type, perioperative complications, graft and patient survival, recurrence of hepatic or metabolic decompensation, and standardized neurological and multisystem follow-up. Until such data are available, successful individual cases show that transplantation is clinically possible but cannot determine its optimal timing or expected benefit for other patients.

### 4.6. Methodological Quality and Research Priorities

Important limitations were observed across the included study designs. These included incomplete reporting of INR and hepatic encephalopathy, difficulty determining whether historical episodes fulfilled PALF criteria, insufficient description of recovery between episodes, incomplete or selective reporting of transplantation outcomes, short or inconsistent follow-up, and possible duplication of patients across publications. These problems were particularly evident in individual case reports and retrospective disease cohorts. Because the review did not include a formal GRADE assessment, the available evidence should not be assigned formal certainty ratings. Nevertheless, confidence in most disorder-specific conclusions concerning prognosis, treatment, and liver transplantation remains limited.

Further progress will require a prospective international registry using uniform definitions of acute liver failure, recurrent episodes, and recovery between episodes. Pseudonymized patient identifiers and transparent reporting of participating cohorts would reduce the risk of counting the same patient more than once. A core outcome set should include the number and timing of episodes, precipitating factors, peak INR, the presence and grade of hepatic encephalopathy, relevant biochemical findings, the extent of recovery between episodes, evidence of chronic liver disease, neurodevelopmental outcome, renal and endocrine involvement, liver transplantation, graft and patient outcomes, and survival. For newly associated genes, experimental studies should use liver-relevant models and examine triggers observed in patients rather than relying exclusively on findings from fibroblasts. Prospective clinical and experimental data of this kind would allow future classifications and recommendations to be based on more reliable disorder-specific evidence.

### 4.7. Limitations

This review is limited by the rarity and clinical heterogeneity of the included disorders and by the predominance of case reports and small retrospective series. Publication bias is likely because severe, unusual, or successfully treated cases may be more likely to be reported. Although no language restriction was applied at the database-search stage, inaccessible non-English full texts may have resulted in the omission of relevant evidence. Definitions of acute liver failure, recurrent acute liver failure, recurrent liver injury, and acute deterioration in the presence of chronic genetic liver disease varied across studies. Some publications included partially overlapping cohorts, and patient-level follow-up after liver transplantation was often incomplete. Embase, Scopus, unpublished data, and trial registries were not searched, and the review protocol was not registered prospectively. These limitations preclude reliable, pooled estimates of prognosis or treatment effect and do not permit formal evidence-graded recommendations. The review should therefore be interpreted as a structured synthesis of the available evidence and a clinically oriented framework for diagnosis and decision-making.

## 5. Conclusions

Recurrent acute liver failure is a clinical presentation shared by several inherited disorders rather than a single diagnosis. The five-group framework proposed in this review is intended to support clinical recognition and differential diagnosis. It represents an author-developed organization of disorders identified in the available literature and should not be interpreted as an exhaustive or formally validated classification of genetic causes of recurrent liver failure. The evidence is derived predominantly from case reports, small case series, and retrospective cohorts in which definitions of acute liver failure, recurrence, and recovery between episodes were inconsistent. Evidence concerning liver transplantation is additionally limited by selection of reported cases, overlapping patient populations, incomplete follow-up, and preferential reporting of unusual or successful outcomes. An inherited cause should be considered during the first unexplained episode of severe acute liver dysfunction rather than only after recurrence. Early metabolic sampling and timely genomic testing may shorten the diagnostic process, particularly when initial biochemical evaluation is nondiagnostic, extrahepatic features suggest a multisystem disorder, or liver transplantation is being considered. Available studies support the diagnostic value of genomic testing but do not yet demonstrate improved survival or other clinical outcomes. Decisions regarding disease-specific treatment and liver transplantation should integrate the severity and reversibility of the acute episode, the course between episodes, the expected natural history, and the extent and prognosis of extrahepatic disease. Because robust comparative outcome data are lacking, these decisions should be individualized by multidisciplinary teams in centres experienced in paediatric acute liver failure, inherited metabolic disease, and liver transplantation.

## Figures and Tables

**Table 1:** Author-proposed staged diagnostic framework for a child with acute or recurrent liver failure.

Purpose and Qualifications	Recommended Investigations	Diagnostic Stage
Assess PALF severity and evolving complications. Repeat neurological examination and laboratory tests according to trajectory—often at least twice daily initially. Give parenteral vitamin K when deficiency is plausible, but avoid routine plasma solely to normalize INR.	Blood group and antibody screen; full blood count with differential and smear; PT/INR, aPTT and fibrinogen; glucose; sodium, potassium, chloride, bicarbonate, calcium, magnesium and phosphate; blood gas; lactate; ammonia; AST, ALT, GGT, alkaline phosphatase; total/direct bilirubin; albumin; urea, creatinine, creatine kinase and lipase; urinalysis.	1. Immediate severity and serial monitoring
Exclude hepatic/portal vascular obstruction, cardiac failure and acute circulatory causes. Imaging should be performed early and must not delay stabilization.	Urgent liver ultrasonography with Doppler. Add ECG and echocardiography when shock, cardiac disease or congestive hepatopathy is possible. Consider dedicated vascular imaging when thrombosis, Budd–Chiari syndrome or tumour infiltration is suspected.	2. Diagnostic imaging and vascular/cardiac assessment
PCR is preferred for suspected disseminated infection in neonates and immunocompromised children. Obtain samples before treatment when feasible, but do not delay empiric acyclovir when neonatal or disseminated HSV is suspected. Broad fixed viral panels should not replace age- and exposure-based selection.	Blood and urine cultures when infection is suspected. Neonates/young infants: HSV PCR, enterovirus and adenovirus testing; CMV where appropriate. Older children: HAV IgM, HBV markers, HCV testing, HEV and EBV/CMV as indicated. Add respiratory, stool, whole-blood or other samples according to phenotype, geography, immune status and exposure.	3. Microbiological diagnostics—age and exposure directed
Exposure history may be incomplete and a late paracetamol concentration may be undetectable. Start NAC promptly when potentially toxic paracetamol exposure is suspected and involve a poison centre when appropriate.	Measure paracetamol concentration in unexplained PALF or whenever exposure is possible. Review prescription, over-the-counter, herbal and recreational substances. Add salicylate, valproate or targeted assays when indicated; test for amanitin when mushroom poisoning is suspected and testing is available.	4. Drugs and toxins
Autoimmune serology may be negative early and HLH criteria can evolve. Interpret ferritin, cytopenias and coagulation findings in clinical context.	Serum IgG; ANA, smooth-muscle, liver–kidney microsomal and soluble liver antigen antibodies. If HLH is suspected: ferritin, triglycerides, fibrinogen, soluble interleukin-2 receptor, NK-cell function/degranulation and bone-marrow assessment when indicated. In neonates consider gestational alloimmune liver disease using specialist-directed iron studies, alpha-fetoprotein and appropriate imaging/pathology.	5. Immune and inflammatory causes
Collect before prolonged protein restriction, renal replacement therapy, transfusion, or initiation of disease-specific metabolic treatment when feasible, but never delay stabilization, glucose delivery, or ammonia-lowering treatment. A normal interval sample does not exclude a genetic disorder.	Plasma amino acids; acylcarnitine profile; total/free carnitine; urine organic acids and orotic acid; urine amino acids for homocitrulline when indicated; blood/plasma ketones; lactate and pyruvate when interpretable; succinylacetone; stored plasma, urine and EDTA blood.	6. Critical metabolic samples—collect early
Age modifies pre-test probability but does not impose an absolute boundary. Red-cell enzyme assays may be unreliable after transfusion. Ceruloplasmin alone neither confirms nor excludes Wilson disease in ALF.	Neonate/young infant: galactosaemia, tyrosinaemia type I, mitochondrial hepatopathy, fatty-acid oxidation disorders, urea-cycle disorders and lysosomal acid lipase deficiency. Infant/toddler: additionally consider hereditary fructose intolerance and congenital disorders of glycosylation. Older child/adolescent: prioritize Wilson disease, mitochondrial/FAO and urea-cycle disorders according to phenotype. Use GALT testing, succinylacetone, copper/haemolysis assessment, enzyme assays and targeted biochemical studies as appropriate.	7. Age- and phenotype-directed metabolic/genetic tests
Consider during the first unexplained severe episode, especially in critically ill infants, recurrent disease, multisystem phenotypes or time-critical transplant decisions. Observational cohorts support diagnostic yield, but outcome benefit remains unproven. Negative exome sequencing may require genome, RNA, structural-variant, protein/enzyme or functional studies.	Rapid trio exome or genome sequencing with copy-number analysis; mitochondrial DNA analysis or a validated rapid comprehensive panel when indicated. Retain DNA and relevant biological samples.	8. Rapid genomic testing (evidence-informed)
Define extrahepatic prognosis and transplant candidacy. Histology in genetic RALF may be nonspecific. Biopsy must not delay genomic diagnosis or urgent treatment; consider a transjugular approach when clinically important coagulopathy is present.	Ophthalmological, neurological and developmental assessment; EEG or brain imaging; cardiac, respiratory, skeletal, immune or haematological evaluation; enzyme/protein assays; RNA and functional studies. Liver or other-tissue biopsy only when expected to change management and technically safe.	9. Specialist and advanced investigations

**Table 2:** Comparative clinical, biochemical, and transplant-related features of inherited disorders associated with recurrent acute liver failure.

Disorder; Gene; Inheritance	Hepatic Presentation and Triggers	Biochemical Clues	Characteristic Extrahepatic Features	Role of Liver Transplantation	Supporting Evidence
OTC deficiency; *OTC*; X-linked	Neonatal hyperammonemic decompensation is typical in severely affected boys; girls and patients with partial deficiency may develop acute or recurrent hepatocellular injury and coagulopathy. Infection, fasting, catabolism, or increased protein intake may precipitate episodes [9].	Hyperammonemia, increased glutamine, low or low-normal citrulline, and increased urinary orotic acid; orotic acid excretion may decrease after protein restriction.	Variable neurocognitive consequences of hyperammonemia; protein aversion or episodic vomiting may be present.	Restores hepatic ureagenesis and prevents further hyperammonemic crises. Consider before irreversible neurological injury in severe neonatal-onset disease or recurrent, medically uncontrolled decompensation; established brain injury is not reversed.	Disease-specific observational evidence; strict RALF subset variably documented
HHH syndrome; *SLC25A15*; autosomal recessive	Acute liver failure or recurrent hepatic decompensation may follow infection, fasting, catabolism, or protein intake [12].	Hyperornithinemia, hyperammonemia, and urinary homocitrulline; the complete triad may not be evident in every sample.	Protein aversion, recurrent vomiting, pyramidal signs, spasticity, cognitive impairment, and white-matter abnormalities.	May prevent further hepatic hyperammonemic crises. Evidence is observational; established neurological injury is not reversed, and neurological progression cannot be assumed to be liver-dependent.	Disease-specific observational evidence; strict RALF subset variably documented
TRMU deficiency; *TRMU*; autosomal recessive	Predominantly infantile acute liver failure, usually confined to the first year of life; lactic acidemia is common. Many survivors have no recurrent liver failure after infancy [15].	Lactic acidosis, hypoglycemia, coagulopathy, and variable aminotransferase elevation. Molecular confirmation is essential.	Possible developmental, neurological, cardiac, muscular, or respiratory involvement, although the phenotype may remain liver-predominant.	Because *TRMU*-associated ALF is frequently reversible and largely confined to infancy, LTx should be individualized. Reserve transplantation for irreversible deterioration despite optimal supportive and cysteine-source treatment, with explicit assessment of extrahepatic disease [15].	Case reports/small series and disease cohorts; mixed strict/contextual evidence
Hepatic DLD deficiency; *DLD*; autosomal recessive	Recurrent infection- or fasting-triggered vomiting, hypoglycemia, hepatocellular injury, coagulopathy, and liver failure [17].	Lactic acidosis and hypoglycemia during crises; acylcarnitines and urinary organic acids may be abnormal during decompensation but normal between episodes.	Variable neurological, muscular, and cardiac involvement.	Only one recipient was identified in the largest hepatic cohort; despite graft engraftment, systemic recovery was poor and the patient died. LTx is not established preventive therapy and should be considered only as rescue treatment after careful assessment of extrahepatic mitochondrial disease [17,18].	Case reports/small series and disease cohorts; mixed strict/contextual evidence
ILFS1; *LARS1*; autosomal recessive	Fever-triggered recurrent aminotransferase elevation, coagulopathy, and acute liver failure, usually beginning in infancy or early childhood [19].	No specific biomarker; hypoalbuminemia and microcytic anemia are common. Diagnosis requires molecular testing.	Growth restriction, hypotonia, seizures, neurodevelopmental abnormalities, and encephalopathic episodes that may resemble metabolic stroke.	One reported recipient had no further hepatic decompensation during 7 years of follow-up, but poor growth, developmental disability, and epilepsy persisted. LTx may control liver crises but cannot be expected to correct multisystem disease [19].	Case reports/small series and disease cohorts; mixed strict/contextual evidence
*IARS1*-related disease; *IARS1*; autosomal recessive	Hepatopathy is usually part of a multisystem phenotype; infection-associated recurrent liver failure has been reported [6].	No specific biochemical marker; aminotransferase elevation, coagulopathy, or hypoalbuminemia may occur during episodes.	Growth restriction, neurodevelopmental disability, hypotonia, and occasionally skeletal abnormalities.	Evidence is insufficient for a disease-specific recommendation. Because the phenotype is multisystemic, candidacy should be determined by the severity and expected progression of extrahepatic disease.	Disease-specific observational evidence; strict RALF subset variably documented
Interstitial lung and liver disease; *MARS1*; autosomal recessive	Variable hepatic dysfunction or liver failure, often in a broader multisystem presentation [6].	No disease-specific routine biomarker; molecular testing establishes the diagnosis.	Interstitial lung disease is the most important diagnostic clue and may accompany or precede liver involvement.	Disease-specific evidence is insufficient. The presence and severity of interstitial lung disease are central to transplant candidacy and expected outcome.	Disease-specific observational evidence; strict RALF subset variably documented
Wolcott–Rallison syndrome; *EIF2AK3*; autosomal recessive	Recurrent hepatitis or acute liver failure, commonly precipitated by infection or physiological stress; liver disease is a major determinant of mortality [21].	No specific metabolic marker for the hepatic episodes; permanent neonatal or early-infancy diabetes is the key diagnostic clue.	Epiphyseal dysplasia, growth impairment, renal dysfunction, hypothyroidism, neutropenia, and recurrent infection may occur.	Isolated LTx and combined liver–pancreas transplantation have been reported in selected patients. Potential benefit must be weighed against diabetes, skeletal disease, infection risk, and other systemic manifestations [21,22].	Disease-specific observational evidence; strict RALF subset variably documented
*NBAS*-associated disease; *NBAS*; autosomal recessive	Fever-triggered recurrent hepatocellular crises ranging from aminotransferase elevation to PALF; substantial recovery between attacks is typical, and crisis frequency often decreases with age [6,28].	Very high aminotransferase activities and variable coagulopathy; no specific biomarker. Interval liver tests may normalize. Molecular testing is required.	Short stature, skeletal abnormalities, optic atrophy, Pelger–Huët anomaly, hypogammaglobulinemia, NK- and B-cell dysfunction, and diabetes in some patients.	May prevent recurrent ALF, but post-transplant morbidity and mortality are substantial in published cohorts. Consider for non-recovering ALF or recurrent life-threatening crises despite optimized emergency care; skeletal, neurological, ophthalmological, immune, and endocrine disease may persist [28,34,35].	Case reports and overlapping retrospective cohorts
ILFS3; *RINT1*; autosomal recessive	Fever-associated recurrent acute liver failure with partial or complete biochemical recovery between episodes; available cohorts remain very small [6].	Marked aminotransferase elevation and coagulopathy during crises; no specific biochemical marker.	Short stature, vertebral abnormalities, kyphosis, nervous-system involvement, optic abnormalities, and occasional cytopenias or immune abnormalities.	Experience is limited to very few recipients. LTx may prevent liver-derived decompensation, but no disease-specific threshold or reliable estimate of long-term extrahepatic outcome can be defined [36].	Case reports/small series and disease cohorts; mixed strict/contextual evidence
CALFAN syndrome; *SCYL1*; autosomal recessive	Recurrent low-GGT cholestasis and fever-triggered liver dysfunction; some patients develop PALF, fibrosis, or progressive chronic liver disease [6].	Conjugated hyperbilirubinemia with inappropriately low or normal GGT; aminotransferase elevation and coagulopathy vary with disease stage.	Peripheral neuropathy, ataxia, tremor, developmental disability, optic atrophy, and skeletal abnormalities; neurological disease may be progressive.	Reported recipients have generally achieved durable hepatic control, whereas neurological disease could persist or progress. Consider for non-recovering ALF or progressive chronic liver disease only after explicit neurological and functional assessment [37,38].	Case reports/small series and disease cohorts; mixed strict/contextual evidence
*AARS1*-related RALF; *AARS1*; autosomal recessive	Infection- or fever-triggered recurrent acute liver failure with complete interval recovery; evidence currently limited to one patient.	No specific biochemical marker; hypoglycemia, coagulopathy, marked aminotransferase elevation, and occasional mild lactic acidosis may occur.	Microcephaly, growth restriction, developmental delay, hypotonia, and neuroimaging abnormalities may accompany liver disease.	No reported liver-transplantation experience for the RALF phenotype; systemic neurological prognosis must be considered.	Single-patient case report
*TRPM7*-associated RALF; *TRPM7*; autosomal recessive	Viral infection-associated recurrent PALF with rapid progression and potentially high early mortality.	No validated biomarker; consider serum magnesium while recognizing that hypomagnesemia was reported in only one patient and is not established as a diagnostic requirement.	Phenotype remains incompletely defined; chronic cholestatic or fibrotic changes were present in some reported patients.	The role of liver transplantation is unknown; evidence is limited to five patients and three early deaths.	Five-patient case series with functional studies

## Data Availability

All data analyzed in this systematic review were derived from published reports cited in the manuscript. Published liver transplantation experience is summarized in Supplementary Table S1, methodological quality and risk-of-bias appraisal in Supplementary Table S2, and database strategies and strict-versus-contextual evidence in Supplementary Table S3. The completed PRISMA 2020 checklist is supplied separately. No new individual-level data were generated.

## Supplementary Materials

The following supporting information can be downloaded at: https://www.mdpi.com/article/10.3390/genes17101225/s1. Table S1: Published liver transplantation experience and evidence category [15,18,19,28,35,36,37,38,48,49]; Table S2: Methodological quality and risk-of-bias appraisal; Table S3: Database search strategies and quantitative separation of strict RALF from contextual evidence; and the completed PRISMA 2020 checklist [48].
