# Supplementary material for: Recurrent Acute Liver Failure in Children: A Systematic Review of Genetic Etiologies, Diagnostic Clues, and Liver Transplantation
Source: Genes (Basel). 2026 Oct 2;17(10):1225. doi: 10.3390/genes17101225 (PMC13704189; doi:10.3390/genes17101225)
Supplement: Supplementary file 1 [file genes-17-01225-s001.zip › S1.pdf]

Supplementary Table S1. Published liver transplantation experience in genetic disorders associated with recurrent acute liver failure

Part A presents recipients for whom a distinct clinical course could be traced in the primary report or an explicit long-term case follow-up. Part B presents aggregate cohorts when individual recipient data were not published. Reports were cross-checked to reduce duplicate counting; aggregate cohorts may overlap with individual reports and must not be summed.

Clinical context	Disorders	Interpretation
Established option in appropriately selected patients	OTC deficiency; selected other urea-cycle disorders	Broader disease-specific experience supports prevention of recurrent hyperammonaemic crises; timing should precede irreversible neurological injury.
Individualized consideration	NBAS, SCYL1, RINT1, LARS1	Published experience supports control of hepatic crises, but survival benefit and optimal timing remain uncertain; extrahepatic prognosis must be considered.
Progressive or non-recovering liver failure despite appropriate treatment	TRMU; hepatic DLD deficiency; EIF2AK3/Wolcott–Rallison syndrome	Spontaneous recovery or multisystem disease may limit benefit. Decisions require careful assessment of reversibility and systemic prognosis.
Insufficient disorder-specific evidence	IARS1, MARS1, AARS1, TRPM7 and other emerging associations	No disorder-specific transplantation recommendation can be made; multidisciplinary assessment is required.

The clinical interpretation reflects the strength and limitations of the available evidence rather than a formal grading system. Liver transplantation is an established option in selected severe urea-cycle disorders, whereas experience in other genetic causes of recurrent acute liver failure is based mainly on individual cases and small retrospective series. Successful transplantation in an isolated patient demonstrates feasibility but cannot establish comparative benefit or optimal timing.

Part A. Individually traceable recipients

Reference / patient	Disease / gene	Pre-transplant course	Age and indication for LTx	Outcome	Follow-up after LTx
Albar et al., 2021 [35]	NBAS-associated disease; female	First PALF at 4 y; ≥3 fever-associated episodes	Age NR; cumulative morbidity from recurrent PALF; diagnosis established after LTx	No recurrent ALF; functioning allograft; clinically well	6 y
Li et al., 2023 [49]; patient 1	NBAS-associated disease; male	11 ALF episodes	3.5 y; transplanted during recovery from ALF	Liver function normalized within 1 mo; no transplant-related complications reported	Duration NR
Li et al., 2023 [49]; patient 2	NBAS-associated disease; male	2 ALF episodes	2.3 y; acute ALF with severe encephalopathy and respiratory failure	Survived; secondary epilepsy, intellectual disability and movement disorder; transient aminotransferase elevation	Duration NR
Li et al., 2023 [49]; patient 3	NBAS-associated disease; female	4 ALF episodes	2.0 y; acute ALF	Died from severe infection within 1 mo after LTx	<1 mo
Cousin et al., 2019 [36]; proband 3	RINT1 deficiency; female	First ALF at 3 y; 4 fever/illness-associated episodes	8 y; recurrent severe ALF (reported peak INR >11)	Post-transplant outcome not described in sufficient detail in the primary report	NR
Lenz et al., 2018 [37]; F3:II.4	SCYL1 deficiency (CALFAN); sex NR	First crisis in infancy; 4 crises	23 mo; recurrent ALF with stage 3–4 bridging fibrosis and nodularity	No further hepatic decompensation; satisfactory graft outcome	8 y
Schmidt et al., summarized by Youssef et al., 2023 [38]	SCYL1 deficiency (CALFAN); sex NR	Infantile ALF; exact episode count NR	21 mo; severe recurrent liver disease	No rejection or postoperative complication reported	11 y
Schmidt et al., summarized by Youssef et al., 2023 [38]	SCYL1 deficiency (CALFAN); female	Infantile ALF; exact episode count NR	7 mo; ALF	Functioning graft; later neurological disease; incisional hernia and cytomegalovirus colitis reported	9 y; patient reported at age 17 y in later follow-up
Youssef et al., 2023 [38]	SCYL1 deficiency (CALFAN); male	Recurrent PALF in infancy; hepatic crises ceased by 2 y; later chronic liver deterioration	20 y; recurrent jaundice/chronic hepatic deterioration	Functioning graft; biliary stricture treated; no rejection; neurological manifestations persisted	3 y
Lenz et al., 2020 [19]; LARS1-09	LARS1 deficiency (ILFS1); sex NR	Severe ALF in early infancy	4 mo; severe ALF	No further hepatic decompensation; albumin and anemia improved; persistent poor growth, developmental disability and epilepsy	7 y

Reference / patient	Disease / gene	Pre-transplant course	Age and indication for LTx	Outcome	Follow-up after LTx
Mihaljević et al., 2024 [18]	DLD deficiency; individual patient	Fulminant liver failure; recurrence history NR	Age NR; fulminant, irreversible liver failure	Good graft engraftment but poor systemic recovery; death from sepsis shortly after LTx	Short-term; exact duration NR

Part B. Aggregate transplant cohorts

Disease(s)	Source	Recipients	Age at LTx	Transplant context	Outcome and follow-up
NBAS-associated disease	Peters et al., 2025 [28]	16	Median 2.15 y (0.08–5 y)	All transplanted for ALF; two required retransplantation	Median 5 y (0–14 y); no recurrent ALF; complications in 9/16; 3/16 deaths; 5-y survival 70% among 10 evaluable recipients
TRMU deficiency	Vogel et al., 2023 [15]	11	Median 4 mo	4 during first ALF, 6 after recurrent ALF, 1 at 11 y for hepatoblastoma	Individual follow-up incompletely reported; 2 deaths after LTx; transplantation was not associated with improved overall survival
NBAS, RINT1, SCYL1 and LARS1 combined	Sutton et al., 2025 [48]	18	Median 2.3 y (4 mo–5.6 y)	11 NBAS, 5 SCYL1, 1 RINT1 and 1 LARS1 recipients	No recurrent ALF after LTx; 2 post-transplant deaths; follow-up heterogeneous and incompletely reported

Abbreviations and limitations: ALF, acute liver failure; CALFAN, low gamma-glutamyl transferase cholestasis, acute liver failure and neurodegeneration; DLD, dihydrolipoamide dehydrogenase; ILFS1, infantile liver failure syndrome type 1; LTx, liver transplantation; NR, not reported; PALF, paediatric acute liver failure; RALF, recurrent acute liver failure. Published transplantation series in OTC deficiency, other urea-cycle disorders and Wolcott–Rallison syndrome include many recipients transplanted for metabolic instability or chronic disease without a clearly documented RALF phenotype; they were therefore not converted into patient-level RALF rows.

Key sources: Albar et al., ACG Case Rep J. 2021;8:e00645; Li et al., Zhonghua Er Ke Za Zhi. 2023;61:66–69; Cousin et al., Am J Hum Genet. 2019;105:108–121; Lenz et al., Genet Med. 2018;20:1255–1265; Lenz et al., Genet Med. 2020;22:1863–1873; Youssef et al., Case Rep Hepatol. 2023:3010131; Mihaljević et al., JIMD Rep. 2024;65:435–443; Vogel et al., Genet Med. 2023;25:100314; Peters et al., Liver Int. 2025;45:e70146; Sutton et al., Liver Int. 2025;45:e70412.
