# Supplementary material for: Recurrent Acute Liver Failure in Children: A Systematic Review of Genetic Etiologies, Diagnostic Clues, and Liver Transplantation
Source: Genes (Basel). 2026 Oct 2;17(10):1225. doi: 10.3390/genes17101225 (PMC13704189; doi:10.3390/genes17101225)
Supplement: Supplementary file 1 [file genes-17-01225-s001.zip › S2.pdf]

# Supplementary Material

*Recurrent Acute Liver Failure in Children: A Systematic Review of Genetic Etiologies, Diagnostic Clues, and Liver Transplantation*

Patryk Lipiński, Piotr Socha and Irena Jankowska

## Table S2. Methodological quality and risk-of-bias appraisal

The included clinical evidence was appraised with the Joanna Briggs Institute design-specific tools. Because the evidence comprises heterogeneous case reports, case series, and retrospective cohorts, no single numerical score was used across designs. The table summarizes the appraisal features that most directly affect interpretation of recurrent acute liver failure, treatment, and transplantation outcomes.

| Evidence body | Predominant designs | Main strengths | Principal risk-of-bias concerns | Implication for interpretation |
|---|---|---|---|---|
| Strict RALF reports | Case reports and small case series | Episode-level clinical descriptions; molecular confirmation in most recent reports | Incomplete historical INR/encephalopathy data; variable proof of interval recovery; selective publication of severe or unusual cases | Supports identification of possible genetic causes, but not reliable estimates of frequency, prognosis, or treatment effect |
| Disease-specific natural-history cohorts | Retrospective multicenter or single-center cohorts | Broader phenotypic range and longer follow-up than isolated reports | Not every participant met the strict RALF definition; referral and survival bias; missing episode-level variables; possible overlap between publications | Used as contextual evidence only and not pooled with strict-RALF denominators |
| Urea-cycle disorders | Guidelines, cohorts, and selected RALF reports | Established biochemical diagnosis and substantial disease-specific clinical experience | RALF is not the dominant phenotype in all reports; neurological outcomes and liver outcomes may be reported together | Management principles are better established than RALF-specific prognostic estimates |
| Energy-metabolism disorders | Case series and retrospective disease cohorts | Genotype-confirmed cohorts available for TRMU and hepatic DLD deficiency | Spontaneous recovery, heterogeneous treatment, incomplete extrahepatic follow-up, and very small transplant samples | Treatment and transplantation conclusions remain low certainty and disease specific |
| Aminoacyl-tRNA synthetase and related disorders | Case reports and small multisystem cohorts | Detailed genotype and phenotype descriptions | Few qualifying recurrent-PALF cases; substantial phenotype heterogeneity; no comparative treatment studies | Evidence supports diagnostic consideration, not generalized treatment or transplantation recommendations |
| AARS1 and TRPM7 | Single discovery reports with very small patient numbers | Molecular findings accompanied by experimental support | No independent replication, uncertain penetrance and spectrum, limited surveillance and outcome data | Emerging associations; variants require rigorous classification and phenotype concordance |
| Vesicular-trafficking disorders | Case reports, case series, and larger retrospective cohorts | Recognizable fever-associated phenotype; multicenter data for NBAS-associated disease | Overlapping cohorts, inconsistent RALF definitions, incomplete long-term and recipient-level follow-up | Supports a strong diagnostic association; estimates of treatment and transplant benefit remain vulnerable to bias |
| Liver transplantation evidence | Retrospective recipient series and individual-patient reports | Documents absence or recurrence of hepatic crises after transplantation in selected disorders | Confounding by indication, survivor selection, duplicate patients, heterogeneous follow-up, incomplete extrahepatic outcomes, no untreated comparator | Cannot establish actuarial survival or causal efficacy; decisions must remain individualized |

Abbreviations: DLD, dihydrolipoamide dehydrogenase; JBI, Joanna Briggs Institute; RALF, recurrent acute liver failure; TRMU, tRNA 5-methylaminomethyl-2-thiouridylate methyltransferase.
