# Supplementary material for: Recurrent Acute Liver Failure in Children: A Systematic Review of Genetic Etiologies, Diagnostic Clues, and Liver Transplantation
Source: Genes (Basel). 2026 Oct 2;17(10):1225. doi: 10.3390/genes17101225 (PMC13704189; doi:10.3390/genes17101225)
Supplement: Supplementary file 1 [file genes-17-01225-s001.zip › S3.pdf]

Supplementary Table S3. Search strategies and quantitative interpretation of strict RALF evidence

Recurrent Acute Liver Failure in Children: A Systematic Review of Genetic Etiologies, Diagnostic Clues, and Liver Transplantation

Patryk Lipiński, Piotr Socha and Irena Jankowska

Part A. Database searches

Formal review cutoff: 2 September 2026. The formal PubMed/MEDLINE export contained 78 records and the supplied Web of Science Core Collection export contained 50 records, yielding 128 records before deduplication. Thirty duplicates were removed and 98 unique records entered screening, as reported in the PRISMA flow diagram. A reproducibility check performed on 23 September 2026 returned 92 PubMed records for the same date-limited strategy, reflecting records indexed or retrospectively tagged after the original export; this later count was not substituted into the completed study-selection dataset.

Database	Strategy / source	Records
PubMed/MEDLINE	(("acute liver failure"[Title/Abstract] OR "fulminant hepatic failure"[Title/Abstract] OR "acute hepatic failure"[Title/Abstract]) AND (recurrent[Title/Abstract] OR recurrence[Title/Abstract] OR episodic[Title/Abstract] OR repeated[Title/Abstract]) AND (child*[Title/Abstract] OR pediatric*[Title/Abstract] OR paediatric*[Title/Abstract] OR infant*[Title/Abstract] OR neonat*[Title/Abstract] OR adolescen*[Title/Abstract]) AND (genetic*[Title/Abstract] OR inherited[Title/Abstract] OR hereditary[Title/Abstract] OR metabolic[Title/Abstract] OR monogenic[Title/Abstract] OR mutation*[Title/Abstract] OR variant*[Title/Abstract] OR exome[Title/Abstract] OR genome[Title/Abstract] OR sequencing[Title/Abstract])) AND ("1900/01/01"[Date - Publication] : "2026/09/02"[Date - Publication])	78
Web of Science Core Collection	Phenotype-first topic search corresponding to acute/fulminant liver failure AND recurrence/episodic presentation AND pediatric age AND genetic/inherited/metabolic disease. The supplied export is retained as the auditable record set; the original platform search-history string was not embedded in the export.	50
Combined before deduplication	PubMed plus Web of Science	128
Duplicates	Matched by PMID, DOI, and normalized title	30
Unique records	Entered title/abstract screening	98

Part B. Strict RALF versus contextual evidence

"Minimum reliably classifiable strict RALF" is deliberately conservative. NR indicates that the publication did not permit reconstruction of a non-overlapping patient-level denominator satisfying repeated PALF-level coagulopathy and documented interval recovery. Contextual cohort sizes must not be summed across overlapping publications.

Disorder/gene	Minimum strict RALF reliably classifiable	Contextual evidence	Interpretive qualification
OTC	NR	Selected reports and broader urea-cycle cohorts	Repeated hyperammonemic decompensation is often reported without complete episode-level PALF and interval-INR documentation.
HHH / SLC25A15	NR	Rare case reports and transplantation cohorts	Acute liver failure is uncommon and strict recurrence cannot be reconstructed consistently.
TRMU	NR	14/62 patients reported recurrent liver-failure episodes	The cohort does not provide sufficient episode-level information to classify all 14 as strict RALF.
DLD	NR	52-patient hepatic DLD cohort plus case reports	Recurrent metabolic/hepatic decompensation is described, but strict PALF denominators are not reconstructable.
LARS1	NR	31 patients in the Sutton individual-participant synthesis	The source framework included broader recurrent acute hepatitis/liver-failure phenotypes; not all can be reassigned to the strict definition.
IARS1	NR	Small multisystem cohorts	Insufficient episode-level INR and recovery data.
MARS1	NR	Small multisystem cohorts	Liver disease is variably defined and often not a strict recurrent PALF phenotype.
EIF2AK3	At least 1 directly documented patient	Broader Wolcott–Rallison cohorts	Additional recurrent liver-failure cases lack uniform PALF-level episode documentation.
NBAS	NR as a non-overlapping total	111 patients in Sutton; 230-patient NBAS hepatic cohort	Substantial cohort overlap and inclusion of recurrent hepatitis or incompletely documented episodes prevent a defensible strict denominator.
RINT1	Up to 3 in the discovery report; interval recovery incomplete in some	6 patients in Sutton plus subsequent reports	Reported recurrence is strong, but strict recovery criteria are not documented uniformly.
SCYL1	NR	20 patients in Sutton; CALFAN discovery and follow-up cohorts	Low-GGT cholestasis, ALF and chronic/progressive liver disease coexist; strict recurrence is not uniformly reconstructable.
AARS1	1	Single functionally supported report	One patient met the recurrent PALF phenotype; no replication cohort.
TRPM7	5 reported as recurrent pediatric ALF	Five patients from three families	Small discovery series; episode-level completeness varies and independent replication is lacking.

Key caution: the 168 participants in Sutton et al. (NBAS 111, LARS1 31, SCYL1 20, RINT1 6) form a clinically valuable disease-specific synthesis but should not be presented as 168 patients meeting the stricter operational definition used in the present review [48].
